# Effects of non-minimum wages on health: A narrative literature review of short- and long-run studies using causal inference or longitudinal data in high-income countries

**DOI:** 10.1016/j.pmedr.2026.103402

**Published:** 2026-02-05

**Authors:** J. Paul Leigh, Juan Du

**Affiliations:** a Department of Public Health Sciences, University of California Davis School of Medicine, Davis, CA, USA; b Center for Poverty and Inequality Research, University of California, Davis, USA; c School of Liberal Arts and Sciences, Musashi University, Tokyo, Japan

**Keywords:** Causal inference, Instrumental variables, Social determinants

## Abstract

**Objectives:**

We reviewed studies examining effects of non–minimum wages on health using causal inference or longitudinal data in high-income countries. We excluded studies on direct effects of minimum wages and on analyses using cross-sectional data without causal designs.

**Methods:**

Our review covered studies from public health, epidemiology, social sciences, and statistics, and published between 1974 and November 2025. Searches were conducted in Google Scholar and PubMed and supplemented by reference and citation tracing. We defined short-run (≤2 years) and long-run (≥5 years).

**Results:**

Thirty-eight studies met inclusion criteria: 20 short-run causal analyses, four short-run longitudinal studies, 12 long-run studies using causal or longitudinal methods, and two encompassing both timeframes. Instrumental variable models were most common, although many instruments (e.g., education and work experience) were invalid. Across 20+ health outcomes—most frequently self-rated health, mortality, and work limitations—results were heterogeneous. We identified recurrent methodological limitations and highlight priorities for future research.

**Conclusions:**

Although findings lack consensus, most studies, particularly long-run analyses, report that lower wages are associated with poorer health, consistent with the allostatic load and Friedman's permanent-income hypotheses. These findings carry implications for minimum-wage-and-health studies in which null findings predominate because those are only short run.

## Highlights

• Reviews exist for minimum wages but not for non-minimum wages, i.e. most workers.
• Causal models eliminate reverse causality bias whereby poor health harms wages.
• We find 38 studies: 24 short-run, 12 long-run, and 2 both short- and long-run.
• Stronger evidence finds higher wages improve health in long- than short-run.
• Evidence helps explain predominate null effects found in minimum wage studies.

## 1 Introduction

Falling, stagnating, and slowly growing wages for lower, middle and some upper classes have been features of the US economy for 45 years (Lind, 2023). They have been implicated in economic, social, and political maladies including widening income inequality (Lind, 2023), falling labor's share of national income (Lind, 2023), increasing gaps between wages and productivity (Mishel and Bivens, 2021), falling fertility (Lind, 2023), divorce, anomie (Case and Deaton, 2020), anti-immigrant sentiment (Macdonald, 2021), political polarization (Lind, 2023), and high dudgeon of the non-college educated for the college educated (Sandel, 2020). Low wages have been linked to use of taxpayer-funded assistance like Medicaid, food stamps, and earned-income tax credits (Jacobs et al., 2015) and as justification for tariffs (Trump, 2025). These wage trends have also been implicated in public health maladies including rising Deaths of Despair (Case and Deaton, 2020) --- liver cirrhosis, drug overdoses, and suicide--- and increasing incidence of disability as measured by uptake of Social Security Disability Insurance (Autor and Duggan, 2006). These trends may have indirect health effects through their effects of income inequality. The trends have been identified as major problems needing amelioration in both Republican and Democratic party platforms (Mishel and Bivens, 2021). Finally, there is some evidence that these trends have been afflicting counties in Europe and Canada, albeit not as pronounced as in the United States (Hayter and Weinberg, 2011). Accordingly, our review includes all high-income countries.

There are debates concerning the causes of these wage trends, but they are beyond the scope of our study (Lind, 2023; Mishel and Bivens, 2021). One cause, nevertheless, is relevant. Medical care improves health. But ever-rising medical insurance paid by employers is likely a cause (Case and Deaton, 2020). The more employers pay for insurance the less available for wages.

Apart from studies using correlational analysis like those involving Deaths of Despair and Disability Insurance, we are not aware of studies analyzing the public health implications of these wage trends. A first step is a literature review. Our study focuses on wages rather than income for several reasons in addition to the wage trends. First, income has many sources including capital gains, dividends, interest and government benefits and these may have different health effects. Wages derive from jobs and are earned; they are not government handouts like Temporary Assistance to Needy Families or the Earned Income Tax Credit. There is no stigma attached to wages (Celhay et al., 2025). Many workers, family members, and society assign status to wages: higher wages connote higher status. Researchers argue that higher status leads to better health (Sayre and Conroy, 2024). Second, the literature on income and health is vast and reviews are available (Marmot, 2002). Third, there are wage-specific government policies such as criteria for overtime pay and promoting unions that, in turn, increase wages.

Our review avoids studies on the direct effects of minimum wages for several reasons. First, literature reviews on minimum wages are available (Leigh et al., 2019). Second, the great majority of the workforce earns more than minimum wages. Third, minimum-wage-and-health studies do not consider long-run effects.

We use the term “non-minimum wages” to refer to wages in studies that are not specifically investigating the direct effects of minimum wages and that typically use difference-in-differences or event-study designs. Non-minimum wage studies might include, for example, those that use minimum wages as an instrument along with other instruments in an instrumental-variables analysis. Incidentally, we did not find any study that used minimum wages as the only instrument.

Our focus is on causal or longitudinal analyses in the short- and long-run. Studies using longitudinal data are viewed as stronger designs than cross-sectional data; models using causal inference, e.g. instrumental variables, are viewed as even stronger (Angrist and Pischke, 2009).^.^We are not aware of any literature reviews focusing on causal models or longitudinal data. Most studies in Sayre and Conroy's (Sayre and Conroy, 2024) review use correlational analysis and cross-sectional data. Their conclusions likely overestimate effects of wages on health because correlational, cross-sectional, studies do not account for reverse causality, and it is well-established that poor health can lead to low wages (Smith, 1999). Wage effects may differ in the short- and long-run as we suggest below. Minimum-wage studies, by design, only operate in the short-run and literature reviews report null findings predominate (Leigh et al., 2019). These null findings can be used to argue that minimum wages do not improve worker or family health. But that argument is not persuasive if it ignores the long run.

Our short- and long-run distinction is consistent with Friedman's permanent income hypothesis (Friedman, 1957). In Friedman's formulation, people do not change consumption behavior when facing transitory changes in income, but they do when facing permanent changes involving 3+ years.

Short-run studies investigate whether wages can affect health within months or 1–2 years (that we define at ≤2). For example, people might exercise more if they can afford a gym.

Long-run studies investigate effects over many years (that we define as ≥5). For example, Duleep^S13^ considers individuals' cumulative earnings from 1968 through 1972 to predict death by 1978. One hypothesis is that some health effects are more likely observed over the long- than short-run. Examples include chronic illnesses such as cancer, heart disease, and Alzheimer's, i.e. illnesses that generally require years to develop and can be affected by allostatic load. Allostatic load, or “weathering”, describes the cumulative “wear and tear” on the body and brain caused by chronic stress (Guidi et al., 2021). Schnorpfeil et al (Schnorpfeil et al., 2003) argue that prolonged exposures to job stressors predict hypertension. In this light, wages, especially low wages, may be viewed just like other occupational exposures such as benzene that raise risks for chronic diseases like cancer but only after years of exposure. Acute occupational illnesses, on the other hand, might be more susceptible to short-run effects. But even these, e.g. covid, can be more severe depending on the strength of immune systems that, in turn, can be affected by obesity resulting from years of overeating. Injuries and behaviors like smoking may be more susceptible to short-run effects than illnesses.

## 2 Method

### 2.1 Screening criteria

Screening criteria included: English-language empirical studies published in scientific journals or reputable research outlets (e.g., National Bureau of Economic Research, Social Science Research Network) that investigated effects of wages on health using causal inference models or longitudinal data in the short and long-runs. We defined short-run as ≤2 years and long-run as ≥5 years (no studies we found spanned >2 to <5 years). Causal models included instrumental variables, difference-in-differences, regression discontinuity, event studies, and marginal structural models using propensity scores. For short-run studies, we required evidence of causal models or longitudinal data. For long-run studies, we required longitudinal data but not causal identification. We searched for two types of longitudinal models: (1) those using person-level fixed effects, random effects, or first differences to analyze wage changes, and (2) long-run models without these adjustments. We excluded theses, student papers, and studies from low-and middle-income countries.

There are enormous literatures we excluded: effects of income or income inequality on health, effects of health on wages, effects of wages on job satisfaction, direct effects of minimum wages or the EITC on health, effects of wages on job absenteeism (most do not distinguish between absence for any reason versus absence due only to illness), compensating wages for hazardous work, and short-run correlational studies. We also excluded smaller literatures involving effects of piece-rate or performance pay, effects of wage replacement rates on receipt of Social Security Disability Insurance or workers' compensation benefits (Autor and Duggan, 2006; Loeser et al., 1995), simulations, or effects on medical services or costs. For the latter ---services or costs--- we reasoned that medical insurance and the income effect ---whereby more services are bought regardless of need--- would be confounding factors. In addition, services or costs are not direct measures of health. We did not require that short-run studies contain data on only individuals; as it turned out, however, only short-run studies with data on individuals passed our screening.

### 2.2 Search procedure

We used four approaches in our mechanical search. The first involved a PubMed search with key words (resulting in 223 potential studies; six selected). The second involved searching the Google Scholar citations to what we believe was the first study of effects of wages on health using causal inference (Grossman & Benham)^S17^ (176 potential studies; eight selected). The third involved tracing the Google Scholar citations to and references within these 14 (11 selected) and citations to and references within those studies (six selected). The fourth applied our knowledge of the literature (seven selected); we have been teaching and researching these topics for decades. The search covered literature in social sciences, statistics, public health, epidemiology, and medicine from 1974 to November 2025. See the appendix for the PubMed code and flow chart.

We searched titles, abstracts, keywords, and instances where “wage” appeared as an independent variable in tables of findings. Our search allowed variations on “wage” including “earnings”, “labor income” and “pay” and variations on “health” including “disability”, “work limitations”, various diseases and injuries, and behaviors e.g. smoking, drinking.

Eleven studies appeared in public health or epidemiology or medical journals (e.g. *JAMA, American Journal of Epidemiology*) and 27 in economics or statistics or social science journals (e.g. *Journal of Health Economics*). See appendix S-Table 1.

### 2.3 Classifying findings

We classified findings as statistically significant and beneficial (positive), significant and harmful (negative), mixed, and insignificant (null). We relied on abstracts, Discussions, Conclusions and findings in tables to determine which studies fell into which category. This review has no data therefore ethics concerns do not apply.

## 3 Results

**Fig. 1:**
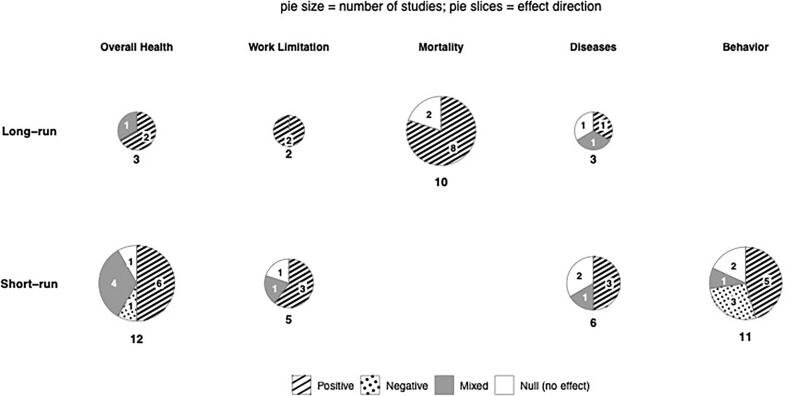
Evidence Map: Health outcomes and horizon for studies of non-minimum wages in high-income countries (1974–2025).

The appendix contains S-Tables 2–4 as well as narrative descriptions of each study pertaining to data/samples, demographics, health outcomes, measurement of wages, methods, instrumental variables, findings, comments and criticisms. Table 1, Table 2, Table 3, Table 4 and Fig. 1 below summarize appendix information.

Table 1 pertains to methods: 1. short-run causal with either cross-sectional or longitudinal data; 2. short-run, non-causal but with longitudinal data; 3. long-run causal with longitudinal data; 4. long-run, non-causal, with longitudinal data. Within these there are sub-categories pertaining to, for example, instrumental variables, longitudinal data with random effects, and event studies. Thirty-eight studies met our criteria: 20 for short-run causal studies, four for short-run non-causal, twelve for long-run, and two for both short- and long-run studies. Appendix S-Table 2 presents an analysis on the overall findings by the four methods. Allowing the last two studies to count twice, twenty-seven studies were in the positive category, two in the negative category, six in the mixed category, and five in the null category. Broken down by time horizon we found 16 positive (62%), two negative, five mixed and three null for the short-run and 11 positive (79%), zero negative, one mixed, and two null for the long-run. The preponderance of evidence suggests wages improve health and this conclusion is especially strong for long-run studies (e.g. no negative findings for long-run studies).

**Table 1:** Included Studies on non–minimum wages and health in high-income countries (1974–2025), categorized by study design and analytical method.

Categories	Authors of studies
I. Short-run causal with cross-sectional or longitudinal data	
Instrumental variables	Asher^S3^, Cai^S4^,Chirikos & Nestel^S6^,, Cottini^S8^, Du & Leigh^S11^, Du & Yagihashi^S11^, Dustmann & Windmeijer^S14,1^, Grossman^S16,1^, Grossman & Benham^S17^, Haveman et al.^S19^, Kim & Leigh^S24^, Lairson et al.^S25^, Lee^S26^, Leigh & Chakalov^S27^, Sedigh et al.^S33^, Sundberg^S34^, Wagstaff^S35^, Xu^S38^
First differences and lagged values of wages as instrumental variables	Dench & Grossman^S9^, Halliday^S18^
Event studies	Rodriguez et al.^S31^, Woo & Shook^S37^
II. Short-run, non-causal, with longitudinal data and person-level random effects	Henseke^S20^, Leigh & Du^S28^, Lindeboom & Kerkhofs^S29^, Nocera & Zweifel^S30^
III. Long-run, causal with longitudinal data and instrumental variables	Ahammer et al.^S1^, Anderson and Burkhauser^S2^, Chapman & Hariharan^S5^, Dustmann & Windmeijer^S14,1^, Ingleby et al.^S21^
IV. Long-run, non-causal with longitudinal data	
First differences with change in wage and person-level random effects	Schmitz^S32,1^
With person-level random effects^2^	Fletcher et al.^S15^, Kezios et al.^S22^
All other long-run, non-causal models with longitudinal data	Christia^S7^, Duggan et al.^S12^, Duleep^S13^, Grossman^S16^^,^^1^, Kezios et al.^S23^, Wolfson^S36^

1 See text for comments on this study.

2 Fletcher et al.^S15^ use general random effects but Kezios et al.^S22^ use random intercepts, a special case of random effects.

The instrumental variables technique was the most popular method for causal inference. Twenty short-run studies (including Halliday (Halliday, 2017) and Dench and Grossman^S9^ who also used first differences) and five long-run studies used instrumental variables. The only other causal model involved event studies. We did not find any studies using difference-in-differences, regression discontinuities, or propensity scores. The next most popular method used longitudinal data with person-level random effects; four short-run and three long-run (including Schmitz (Schmitz, 2016) who also used first differences). We did not find any studies using person-level fixed effects. The third most popular used longitudinal data without fixed or random effects.

Dustmann & Windmeijer^S14^ and Grossman^S16^ enter twice into the table. Dustmann & Windmeijer^S14^ analyze “transitory” and “permanent” changes in wages, terms that we interpret as short-run and long-run and that are identical to Friedman's hypothesis. They find negative effects in the short-run but positive effects in the long-run (appendix S-Table 2). Grossman^S16^ analyzes current “health stock” ---composite measures involving self-assessed excellent-to-poor health combined with number of healthy workdays---- with cross-sectional data, and mortality with longitudinal data. We include “health stock” in our short-run category and mortality in our long-run category. He finds positive effects in the short-run but null effects in the long-run (appendix S-Table 2).

Appendix S-Table 2 provides information on countries. In addition to the US, we included studies from Australia (Cai^S4^), 15 European countries (Ahammer et al.^S1^; Cottini^S8^; Dustman & Windmeijer^S14^; Henseke^S20^; Ingleby et al.^S21^; Lindeboom & Kerkhofs^S29^; Nocera & Zweifel^S30^; Sundberg^S34^; Wagstaff,^S35^) and Canada (Sedigh et al.^S33^; Wolfson^S36^). Counting Grossman^S16^ and Dustmann & Windmeijer^S14^ twice, there were 40 studies. Thirteen-of-40 studies were from countries outside the United States; six-of-22 in the short-run causal column, three-of-four in the short-run non-causal column, three-of-five in the long-run causal column, and one-of-nine in the long-run non-causal column.

Table 2 presents outcomes for short-run studies within positive, negative, mixed, and null groupings. We identified four broad outcome categories (from overall subjective health to behavior) and 11 subcategories (from composite measures to obesity). Outcomes were not measured in the same units across studies. For example, some studies converted self-reported, excellent-to-poor to a binary variable and used logistic regression while others assigned numbers such as 1 for excellent, 2 for very good, and so on, and used ordered probit regression. In addition, there was some variation in measures of wages. While most studies used wages-per-hour, others used wages-per-week, wages-per-month, and annual labor earnings. Most studies focused on one or two outcomes, but some considered three or more, typically specific diseases or conditions e.g. stroke, cancer, hypertension, cognitive function. The two most frequent outcomes were overall subjective health and work limitations.

**Table 2:** Short-Run (≤2 Years) Studies of Non–Minimum Wages and Health in High-Income Countries (1974–2025), by Causal, Longitudinal, and Non-Causal Designs.

Outcome	Positive	Negative	Mixed	Null
I. Overall subjective health				
Composite measures using self-reported excellent-to-poor combined with measures of days in good health, and “health better than others”^1^	Grossman^S16^, Lairson et al.^S25^, Lindeboom & Kerkhofs^S29^, Wagstaff^S35^	Dustmann & Windmeijer^S14^	Nocera & Zweifel^S30^, Sundberg^S34^, Asher^S3^	Dench & Grossman^S9^, Grossman & Benham^S17^
Excellent-to-poor only, no composite, mostly binary but also 1…5, ordinal scale.^2^	Grossman^S16^, Halliday^S18^, Lee^S26^, Fletcher et al.^S15^		Henseke^S20^, Schmitz^S32^	Cai^S4^
II. Work Limitation, functional disability	Chirikos & Nestel^S6^, Haveman et al.^S19^, Lee^S26^		Asher^S3^, Henseke^S20^	Cai^S4^, Dench & Grossman^S9^
III. Diseases and conditions				
Numbers of diseases, injuries or symptoms; composite for many diseases	Cottini^S8^			Cai^S4^, Dench & Grossman^S9^, Grossman & Benham^S17^
Hypertension, heart disease	Leigh & Du^S28^		Henseke^S20^	
Financial anxiety and food insecurity	Woo & Shook^S37^			
IV. Behavior				
Smoking	Du & Leigh^S11^, Leigh & Chakalov^S27^	Xu^S38^		
Exercise	Du & Yagihashi^S10^	Dustmann & Windmeijer ^S14^		Xu^S38^
Drinking				Xu^S38^
Minutes of sleep for insomniacs		Sedigh et al.^S33^		
Number of vehicle crashes	Rodriguez et al.^S31^			
Obesity, body mass index	Kim & Leigh^S24^			

1 There are different measures. For example, Grossman^S16^ combines data on absence from work and excellent-to-poor. See appendix S-Table 4 for other measures.

2 Sometimes excellent-to-poor is treated as binary (excellent = 1) (e.g. Grossman^S16^) and sometimes all ordinal values of excellent-to-poor are included in an ordered logit or probit regression (e.g. Cai^S4^). See appendix S-Table 4.

There was no consensus among any of the outcomes in terms of findings but there were tendencies and inconsistencies. The tendency for numbers of diseases is statistical insignificance because three studies report null findings, only one reports positive findings, and zero in the two other categories. The tendency for studies in the overall health, work limitations and diseases categories combined is for non-negative findings; only one of 18 studies reported negative findings. Composite measures of health and measures involving excellent-to-poor were inconsistent with more null and mixed results than positive results. Results on smoking present sharp contrasts. Two studies (Du and Leigh, 2015; Leigh and Chakalov, 2023) find higher wages lead to lower smoking prevalence while Xu (Xu, 2013) find higher prevalence; no studies find mixed or null effects.

Results in Table 2 can address the hypothesis that short-run effects may more likely be observed for behaviors than for chronic diseases. No clear patterns emerge, however. Within “Numbers of diseases” and “hypertension & heart disease”, two studies report positive effects, one mixed, and three null effects. Within “Behaviors”, five report positive, three negative, and two null.

Many studies analyzed unique outcomes. Some found positive effects, e.g. vehicles crashes, obesity, and financial anxiety while others found null effects e.g. drinking and depression. (While most vehicle crashes do not result in injury, in our view, they measure risky behavior, like smoking).

Some findings indicated higher wages harmed health. Sedigh et al (Sedigh et al., 2017), found higher wages predicted fewer minutes of sleep among people with insomnia and Xu (Xu, 2013) found higher wages predicted more smoking. Asher^S3^ found wage growth, not current wages, harmed subjective health and increased work limitations; her findings for current wages were the opposite (i.e. positive).=

Table 3 presents results for long-run studies. Mortality had the strongest tendency. In eight of ten studies, higher wages significantly predicted lower mortality; none predicted higher mortality; and two found insignificance. For work limitations and functional disability, two studies reported positive findings, and none reported in the other categories. For memory loss, Kezios et al (Kezios et al., 2022) reported positive findings and Schmidt (Schmitz, 2016) reported null findings. Perhaps most importantly, none of the 17 long-run findings were negative.

**Table 3:** Long-Run (≥5 Years) longitudinal studies of non–minimum wages and health in high-income countries (1974–2025), including causal and non-causal analyses.

Outcome	Positive	Negative	Mixed	Null
I.Overall health (excellent-to-poor and days in good health)	Dustmann & Windmeijer^S14^		Schmitz^S32^	
II. Work Limitation, functional disability	Anderson and Burkhauser^S2^, Duleep^S13^			
III. Diseases				
Depression			Schmitz^S32^	
Memory loss, word recall	Kezios et al.^S22^			Schmitz^S^^32^
IV Mortality, length of time to death	Anderson and Burkhauser^S2^, Chapman & Hariharan^S5^, Christia^S7^, Duggan^S12^, Duleep^S13^, Ingleby et al.^S21^, Kezios et al.^S23^, Wolfson^S36^			Ahammer et al.^S1^, Grossman ^S16^

We combined the 13 non-USA countries information in appendix S-Tables 2 and 4 and found six-of-13 in the positive column, two-of-13 negative, three-of-13 mixed, and two-of-13 null. Broken down by time horizon, we found three-of-nine positive, two-of-nine negative, three-of-nine mixed and one-of-nine null within short-run studies and three-of-four positive, zero-of-four negative, zero-of-four mixed, and one-of-four null within long-run studies. Within these 13 non-USA countries there is less evidence that wages improve health either overall or within short-run studies; but there again appears more evidence for the wage-improving-health hypothesis in the long-run studies. Combining both United States and non-United States studies, the only two negative findings were from non-USA studies: Canada (Sedigh et al., 2017) and Germany (Dustmann & Windmeijer^S14^).

The evidence map (Fig. 1) presents summaries of Table 2, Table 3. Combining both short- and long-run findings, 30 are positive, four negative, nine mixed, and nine null. For short run findings, the numbers are 17 positive (50%), four negative, seven mixed, and six null. For long-run, 13 are positive (72%), zero negative, two mixed and three null. We conclude that the preponderance of evidence supports the hypothesis that higher wages improve health, and that this conclusion is especially strong for long-run studies. These overall findings are identical to those above pertaining to the 38 studies. While they are not consistent with the smaller number of non-USA studies for overall health, they are for the smaller number of non-USA long-run studies. The total number of findings (52) exceeds the number of studies (38) because some studies investigate more than one health variable.

The most frequent causal model involved instrumental variables. Appendix S-Table 3 presents information on instruments ranked from most to least popular. Most popular included education, years of work experience, and southern residence. Moderately popular included union membership or coverage, city residence, minimum wages, industry indicators, blue-collar job, and lagged values of wages or health.

Table 4 presents categories of instruments. We judged 16 to be invalid or likely invalid, 12 to perhaps be valid and none to definitely be valid. Most instruments likely affected health or behavior independent of wages. (Most studies were from the 1900s, before investigators paid attention to validity). Education, including parents' educations, and general intelligence predict good health, independent of wages.^S16^ Years of work experience is correlated with age and age affects health. Blue-collar jobs are more hazardous than white-collar jobs. Blacks and unmarried people experience poorer health than whites and married people. Unemployment affects health even among the employed (Miller et al., 2009). Other instruments likely have more validity but could still be questioned as they involve workers' choices, e.g. workers can choose work in large firms in unionized industries. Minimum wages, lagged wages, average wages within occupational categories, and living in states with high union density have promise for validity. But even these have drawbacks e.g. minimum wages do not affect most workers and lagged wages require strict econometric assumptions.

**Table 4:** Instrumental variables used in causal studies of non–minimum wages and health in high-income countries (1974–2025), with key limitations.

Instruments	# of Studies	Limitations
Job-related: work experience, union member, industry, occupation, blue/white collar, part-time, firm size, work organization index, industry unemployment rate	20	Industry, occupation, firm size, and organization index perhaps valid but weak. All others invalid.
Education (own and parents), vocational training, knowledge of software, and intelligence	7	Education and intelligence invalid. Knowledge and training perhaps valid but weak
Residence: city/rural, southern, residence	8	Likely invalid
State variables: minimum wage, union density, unemployment rate, labor force participation rate, occupational wage	5	Unemployment rate invalid. All others perhaps valid but weak
Lagged variables of wages and health	3	Perhaps valid; can only be used in panel data
Demographics (marital status, race) and non-labor income	5	Invalid
Health: Activities of Daily Living and work limitations	2	Invalid

## 4 Discussion

Literature reviews are available on direct (as opposed to indirect, i.e. instrumental variables) effects of minimum wages on health involving causal inference and longitudinal data (Leigh et al., 2019). Minimum wage studies consider only short-run effects, however. Another review covers all wages, but conclusions are questionable as most studies in that review use correlational analyses with cross-sectional data (Sayre and Conroy, 2024) thereby permitting bias from reverse causality: poor health reduces wages. We believe ours is the first review of non-minimum wage studies using causal inference or longitudinal data in short- and long-run settings.

The preponderance of evidence suggests that higher wages improve health, and this is especially true for long-run studies. Short-run effects last months or at most two years; long-run effects last many years or even decades. Our long-run findings are consistent with the allostatic load hypothesis, Friedman's permanent income hypothesis, and viewing low wages as risk factors for chronic rather than acute occupational diseases; our findings also address Kawachi et al.'s (Kawachi et al., 2010) query about “temporary” versus “long-term” income shocks.

Like the reviews of minimum-wage-and-health studies, our studies show numerous health outcomes; there is no single, preferred, health measure. The most consistent results indicated higher wages lead to lower mortality and fewer work limitations.

Table 5 lists implications for future research organized into two sections: Design & Methods and Theory. First, we advocate for quasi-experimental designs. Policy shocks such as increases in minimum wages are excellent examples but, at least in the case of minimum wages, they cannot help with the majority of workers that earn above those minimums and they may not extrapolate to the long-run. The second issue pertains to instrument validity; most are invalid. Recent research on monopsonies suggest measures of labor market concentration might be useful instruments (Azar et al., 2022). The third issue involves extant bias against studying women, minorities, and children. The fourth issue involves attrition and competing-risks bias in mortality studies. People can drop out over time due to health problems and die of diseases that are not being analyzed. No study here accounts for these potential biases but future research should. The remaining issues in Design & Methods require no additional comment. The first Theory issue concerns defining wages. Many studies use weekly, monthly or annual wages divided work hours. But work hours respond to wages along the labor supply curve and work hours have their own effects on health (Bannai and Tamakoshi, 2014). Future research might use actual wage rates on-the-job as provided in the Panel Study of Income Dynamics, the National Longitudinal Surveys, and the Health and Retirement Surveys. The second Theory issue pertains to unemployment. Many long-run studies do not account for unemployment; they simply add-up earnings over many years. But unemployment can have direct effects on health independent of wages^.^ (Miller et al., 2009)

**Table 5:** Methodological gaps and priorities for future research on non–minimum wages and health in high-income countries (1974–2025).

Category	Study/authors
I. Design	
Causal inference, quasi-experimental design, policy shocks	See first three rows of Table 1
Instruments; most not valid; many studies omit tests for validity or strength; labor-market concentration shows promise	See Table 4
Demographics. Many studies include white men only or men only. No studies include children	Anderson and Burkhauser^S2^, AsherS3, Cai^S4^, Chapman & Hariharan^S5^, Duleep^S13^, Dustmann & Windmeijer^S14^, Grossman^S16^, Grossman & Benham^S17^, Haveman et al.^S19^, Lairson^S25^, Lee^S26^, Lindeboom & Kerkhofs^S29^, Schmitz^S32^, Wolfson^S36^, Xu^S38^
Attrition and competing-risks bias in mortality studies.	Most longitudinal studies do not account for attrition or competing risks. Unbalanced designs and person-level fixed effects might help for attrition.
Should sick leave, medical insurance, or doctors-per-capita be covariates?	Almost all studies omit. Exceptions include Grossman & Benham^S17^ and Leigh & Du^S28^ who consider insurance and Wagstaff^S35^ who considers doctors-per-capita.
Wage growth vs level of wages?	Most studies consider only level of wages, not growth. Exceptions include Asher^S3^, Halliday^S18^, Schmitz^S32^.
Should there be adjustments for health at baseline for long-run studies?	All long-run studies without instrumental variables include some measures of health at baseline; no long-run instrumental variables studies do.
Should studies account for other family income as a covariate?	Leigh & Du^S12^ include other family income as a covariate. Most studies omit.
Should studies account for job satisfaction as a covariate?	Grossman^S16^ and Lairson^S25^ include as a covariate. Most studies omit. We believe satisfaction is on pathway from wages to health so appropriate to omit.
Multiple comparisons with, for example, Bonferroni adjustments?	Dustmann & Windmeijer^S14^, Schmitz^S32^, and others have many outcomes and demographic subgroups. No study made multiple comparison adjustments.
Many long-run studies measure death as binary with logistic or probit regressions, not as time-to-death with a Cox regression. Which is better?	These studies use only binary death: Ahammer et al.^S1^, Anderson and Burkhauser^S2^, Christia^S7^, Duggan^S12^, Grossman^S16^.
Should studies account for diminishing returns?	Almost all studies omit. But diminishing returns are frequently found for effects of income on health.

II. Theory	
Wage definition. Definition of wages includes work hours, e.g. weekly or monthly or annual wages divided by weekly or monthly or annual hours. But wages can affect hours and hours can affect health.	Most studies. Few exceptions include Grossman & Benham^S17^, Kezios et al.^S23^ and Lee^S26^ who measure wage rate provided by respondent. Haveman et al.^S19^ treat hours as endogenous. Fletcher et al.^S15^ include cumulative work hours as a covariate.
Unemployment. Studies include people who experience periods of unemployment, especially those using Social Security earnings data; other studies assign the unemployed to the low-wage group. But unemployment can affect health.	Almost all long-run studies. Exceptions include Fletcher et al.^S15^ who include unemployment spells as a covariate and Wolfson^S36^ who includes “interrupted” work history as a covariate.
Job precarity, psychological job stress and effort-control imbalance likely correlate with low wages. Should precarity, stress, or imbalance be accounted for?	Only Kezios et al.^S22,S23^ discuss precarity; only Leigh & Du^S28^ mention job stress.
Pay-for-performance?	No study accounts for pay-for-performance
Should compensating wage differentials for hazardous work be accounted for? How?	Only Dench & Grossman^S9^ explicitly account for compensating wages. But Fletcher et al.^S15^, Haveman et al.^S19^, Henseke ^S20^, Schmitz^S32^ and Sundberg^S34^ include measures of hazards as covariates. Compensating wages may not exist, however.

The epidemiologic and public health literature is replete with studies on negative effects of job precarity, job stress (or strain), and effort-reward imbalance (third issue under Theory) (Schnall et al., 1994; Siegrist, 2002; Utzet et al., 2020). But wages, especially low wages, likely correlate with precarity, stress, and imbalance. In fact, some studies include low wages as part of the definition of precarity. It could be that any correlation between low wages and health masks for the effects of precarity, stress or imbalance.

Our findings have implications for related research. First, reviews of minimum wage studies conclude that null effects are the most frequent findings. Considering our distinction between the short- and long-run, these null findings may have an explanation. Minimum-wage studies look for effects within months or one or two years, but allostatic load typically requires many years. Our findings, when combined with those from minimum wage studies, suggest that wages are more likely to have salubrious effects in the long-run than the short-run. Second, if low wages harm health, then long-term falling wages may help explain falling labor force participation among prime-age workers and rising numbers of people on Social Security Disability rolls (Autor and Duggan, 2006). Third, work precarity, job stress or strain, and effort-reward imbalance might mask for low wages in the epidemiologic and public health literature (Schnall et al., 1994; Siegrist, 2002; Utzet et al., 2020). We are not aware of stress or imbalance studies that account for wages or any precarity studies that separately account for wages. Fourth, work hours might mask for wages along the labor supply curve in the epidemiologic and public health literature. Few epidemiologic or public health studies on hours properly account of wages.

Our review has limitations. First, it is narrative, not systematic. But prior minimum-wage-and-health reviews and the one all-wages review are also not systematic (Sayre and Conroy, 2024; Leigh et al., 2019). We nevertheless explain our selection criteria that rely on Google Scholar and PubMed in detail in the appendix. We did not conduct meta-analyses; Table 2, Table 3 show the difficulty associated with any such attempt. There were too few studies for any given outcome and even within those studies, frequently neither outcomes nor wages were measured in the same units. We did not select “better” and “worse” studies among the 38 that met our criteria; major criticisms appear in Table 5. We nevertheless recommend recent short-run studies by Dench and Grossman^S9^ and Du and Yagihashi (Du and Yagihashi, 2017) and long-run studies by Kezios et al (Kezios et al., 2022; Kezios et al., 2023). Finally, we restricted attention to only high-income countries.

## 5 Conclusion

Falling, stagnating, and slowing growing wages present economic, social, and political concerns; many believe these concerns should extend to health. Researchers have responded with rapidly increasing numbers of minimum-wage-and-health studies; but these apply to only the short run and most of the workforce earns more than minimum wages. Studies on non-minimum wages or simply low wages, or long-run effects, have been lagging. We hope this review will encourage research into the role all wages play in affecting the health of workers and their families.

## CRediT authorship contribution statement

**J. Paul Leigh:** Writing – review & editing, Writing – original draft, Methodology, Formal analysis, Conceptualization. **Juan Du:** Writing – review & editing, Methodology, Formal analysis.

## Declaration of competing interest

The authors declare that they have no known competing financial interests or personal relationships that could have appeared to influence the work reported in this paper.

## Data Availability

No data was used for the research described in the article.

## Appendix A Supplementary data

Appendix Image 1
