# Supplementary material for: Effects of non-minimum wages on health: A narrative literature review of short- and long-run studies using causal inference or longitudinal data in high-income countries
Source: Prev Med Rep. 2026 Feb 5;62:103402. doi: 10.1016/j.pmedr.2026.103402 (PMC12908019; doi:10.1016/j.pmedr.2026.103402)
Supplement: Supplementary file 1 — Appendix [file mmc1.docx]

Appendix to “Effects of non-minimum wages on health: A narrative literature review of short- and long-run studies using causal inference or longitudinal data in high-income countries”

1. PRIMSA-style flow chart
2. Appendix S-table 1. Classification of studies by academic area
3. Appendix S-table 2. Categories of studies by methods and overall findings
4. Appendix S-table 3. Instruments used in instrumental variables analyses
5. Appendix S-table 4. Authors, samples, methods, findings. Note: Grossman (1976) and Dustmann & Windemeijer (2000) appear twice: once in short-run causal and once in long-run causal
6. Long form descriptions of studies. Organized alphabetically and numbered as S1, S2, S3, etc.
7. Studies that, at first, seemed to fit criteria, but were ultimately rejected. Long form descriptions. No separation by short-run or long-run or causal
8. PRIMSA-style flow chart

We used this code on within PubMed: “(wage AND ((health OR mortality OR smoking OR obesity) AND (“instrumental variables” OR “two stage least squares” OR “regression discontinuity” or “difference-in-differences” OR “propensity scores” OR “event study”)) OR (wage AND mortality AND longitudinal).” The first part of the code applies to short-run studies and the second to long-run ones. This code produced 223 entries; we looked at each one. We found four short-run studies ---Du & Leigh (2015), Kim & Leigh (2010), Leigh & Chakalov (2023), Xu (2013)--- and two long-run studies ----Ingleby et al (2021) and Kezios et al. (2023) that fit our criteria.

The same code we used for PubMed yielded 13,200 references within Google Scholar. We did not judge it to be feasible to look at each entry one-by-one. Our alternative approach was to investigate citations to Grossman & Benham (1974). We believe this was the earliest study on the effects of wages on health that uses some causal inference. There were 176 citations. We looked at each one. We discovered eight short-run and no long-run studies that met our criteria. Here are the eight: Cai (2009), Chirikos & Nestel (1984), Dench & Grossman (2019), Grossman (1976), Haveman et al. ( 1994 ), Lairson (1984 ), Lee (1982), Wagstaff (1986).

PRIMSA-style Flow Chart.

| Citations to Grossman & Benham (N= 176 entries) | Code (S-table footnote 1) into PubMed finds 223 entries |
| --- | --- |
| Reasons (and numbers of studies) eliminated | Reasons (and numbers of studies) eliminated |
| No health dependent variable (N = 24) | Study did not include wage as an independent variable (n = 75) |
| Google Scholar reports “CITATION” meaning entry is a citation, not a published study (24) | No causal inference or other qualifying method and short-run study (40) |
| Reverse causality i.e. poor health harms wages (21) | Medical insurance or services was dependent variable (29) |
| No causal inference or other qualifying method and short-run study (20) | Minimum wages only (29) |
| Outside OECD (19) | Reverse causality i.e. poor health harms wages (16) |
| Literature review or essay or theory or thesis or working paper (18) | Outside OECD (15) |
| Entry is in foreign language (17) | Absenteeism or pay-for-performance (10) |
| Duplicates (9) | EITC (5) |
| Study uses income or wealth, not wage (8) | Literature review or essay or theory or thesis or working paper (4) |
| Other (8) |  |
|  |  |
| Eight qualifying studies: Cai (2009), Chirikos and Nestel (1984) , Dench & Grossman (2019), Grossman (1976), Haveman et al. ( 1994 ), Lairson (1984 ), Lee (1982), Wagstaff (1986). All of these are short-run studies except Grossman (1976) that is both short- and long-run. | Six qualifying studies. Four short-run: Du & Leigh (2015), Kim & Leigh (2010), Leigh & Chakalov (2023), Xu (2013); and two long-run: Ingleby et al (2021) and Kezios et al. (2023) |

1. (wage AND ((health OR mortality OR smoking OR obesity) AND (“instrumental variables” OR “two stage least squares” OR “regression discontinuity” or “difference-in-differences” OR “propensity scores” OR “event study”)) OR (wage AND mortality AND longitudinal)

The 14 studies at the bottom of our PRIMSA-style S-table form the first selection list. We then examined the citations to these 14 studies and found 11 more that qualified; we repeated the citation/reference examination on those 11 and found six unique additional studies (after subtracting two PubMed overlapping studies). Finally, we added seven studies with which we were familiar having taught the relevant classes for decades. These seven were: Cottini (2012), Duggan et al. (2008), Fletcher et al. (2010), Halliday (2017), Henseke (2018) , Rodriquez et al. (2006), Sedigh et al. (2017).

II. Appendix S-table 1. Classification of Studies by academic area

| Economics, statistics, social science, and authors | Public health, epidemiology, medical, and authors |
| --- | --- |
| Book chapters, all economics: Dench & Grossman (2019), Grossman (1976), Grossman & Benham (1994), Nocera & Zweifel (1998), Sundberg (1998) | Social Science & Medicine: Ingleby et al. (2021), Lairson et al. (1984), Xu (2013) |
| Journal of Health Economics: Christia (2009), Haveman et al. (1994), Schmidt (2016), Wagstaff (1986) | Journal of Occupational and Environmental Medicine: Kim & Leigh (2010), Leigh & Chakalov (2023) |
| Journal of Human Resources: Anderson and Burkhauser (1986), Chirikos & Nestel (1984), Duleep (1986) | Medical Care: Asher (1984) |
| Economics and Human Biology: Sedigh et al. (2017) | Annals of Epidemiology: Du & Leigh (2015) |
| Health Economics: Fletcher et al. (2010) | American Journal of Epidemiology: Kezios et al. (2022) |
| Journal of the Royal Statistics Society Series A: Ahammer et al. (2017) | Journal of the American Medical Association: Kezios et al. (2023) |
| Manchester School: Cottini (2012) | European Journal of Public Health: Leigh & Du (2012) |
| IMF papers: Duggan et al. (2008) | Journal of Gerontology: Wolfson et al. (1993) |
| SSRN papers: Dustmann & Windmeijer (2000) |  |
| Review of Income and Wealth: Halliday (2017) |  |
| European of Journal of Health Economics: Henseke (2018) |  |
| International Economic Review: Lee (1982) |  |
| Journal of Applied Econometrics: Lindeboom & Kerkhofs (2009) |  |
| Industrial and Labor Relations Review: Rodriguez et al. (2006) |  |
| Journal of Human Behavior and Social Environment: Woo & Shook (2023) |  |
| The Economic Record: Cai (2009) |  |
| Journal of Risk and Uncertainty: Chapman & Hariharan (1994) |  |
| Review of Economics of the Household: Du and Yagihashi(2017) |  |

1. Appendix S-table 2. Categories of studies by methods and overall findings

| Categories | Significant and beneficial (positive) | Insignificant (null) | Significant and harmful (negative) | Mixed^1^ |
| --- | --- | --- | --- | --- |
| I. Short-run causal with cross-sectional or longitudinal data |  |  |  |  |
| Instrumental variables | Chirikos & Nestel (1984), Cottini (2012)(15 European countries), Du & Leigh (2015), Du & Yagihashi (2017). Grossman (1976)^1^, Haveman et al. (1994), Kim & Leigh (2010), Lairson et al. (1984), Lee (1982), Leigh & Chakalov (2023), Wagstaff (1986)(Denmark) | Cai (2009) (Australia) , Grossman & Benham (1974) | Dustmann & Windmeijer (2000)^1^(Germany), Sedigh et al. (2017)(Canada) | Asher (1984), Sundberg (1998)(Sweden), Xu (2013) |
| First differences and lagged values of wages as IVs | Halliday (2017) | Dench & Grossman (2019) |  |  |
| Event studies, but no parallel trends tests | Rodriguez et al. (2006), Woo & Shook (2023) |  |  |  |
| II. Short-run, non-causal, with longitudinal data |  |  |  |  |
| With individual random effects | Leigh & Du (2012), Lindeboom & Kerkhofs (2009)(Netherlands) |  |  | Henseke (2018)(European countries), Nocera & Zweifel (1998)Switzerland) |
| III. Long-run, causal with longitudinal data |  |  |  |  |
| Instrumental variables | Anderson and Burkhauser (1986), Chapman & Hariharan (1994),  Dustmann & Windmeijer (2000)1(Germany), Ingleby et al. (2021)(UK)) | Ahammer et al. (2017)(Austria) |  |  |
| IV. Long-run, non-causal with longitudinal data |  |  |  |  |
| First differences with change in wage and random effects |  |  |  | Schmitz (2016)^1^ |
| With random effects^2^ | Fletcher et al. (2010), Kezios et al. (2022) |  |  |  |
| All other long-run, non-causal models with longitudinal data | Christia (2009), Duggan et al. (2008), Duleep (1986), Kezios et al. (2023), Wolfson (1993)(Canada) | Grossman (1976)^1^ |  |  |

Footnotes. 1. See text for comments on this column or study. 2. Fletcher et al. (2010) use general random effects, but Kezios et al. (2022) use random intercepts which is a special case of random effects. Comment on findings: The six studies in the mixed column have unique findings. Nocera & Zweifel , Schmitz , and Sundberg find some results are insignificant but others significant and beneficial. Xu finds some insignificant and others significant and harmful. Henseke finds mostly insignificant but some significant and both beneficial and harmful. Asher finds significant and beneficial effects for the level of health but significant and harmful for the change in health. Finally, Schmitz finds that earnings level does not significantly predict self-assessed health but earnings growth significantly predicts positive self-assessed health. Further, Schmitz finds earnings level significantly predicts good cognitive functioning whereas earnings growth does not. Schmitz is the only study of these six that is long run. 3. Countries appear in parentheses; if no parenthesis, then USA.

1. Appendix S-table 3. Instruments used in instrumental variables analyses

| Instrument | Study/authors |
| --- | --- |
| Education, in years and binaries | Asher (1984), Chirikos & Nestel (1984), Dustmann & Windmeijer (2000), Lairson et al. (1984), Sedigh et al. (2017), Anderson and Burkhauser (1986), Chapman & Hariharan (1994) |
| Work experience, years | Asher (1984), Cai (2009), Chirikos & Nestel (1984), Grossman (1976), Grossman & Benham (1974), Haveman et al. (1994), Lairson et al. (1984), Lee (1982), Sundberg (1998) |
| Southern residence, binary | Chirikos & Nestel (1984), Grossman (1976), Grossman & Benham (1974), Haveman et al. (1994), Lairson et al. (1984), Lee (1982) |
| Union member or coverage, and percent union, binary and continuous | Cai (2009), Du & Leigh (2015), Grossman & Benham (1974), Leigh & Chakalov (2023), Sedigh et al. (2017) |
| City/rural, SMSA residence, binary | Asher (1984), Cai (2009), Grossman (1976), Grossman & Benham (1974), Lairson et al. (1984), Lee (1982) |
| Minimum wage, continuous | Cottini (2012), Du & Leigh (2015), Du & Yagihashi (2017). Kim & Leigh (2010) |
| Industries, firms, binaries | Sedigh et al. (2017), Xu (2013), Ahammer et al. (2017) |
| Blue- or white-collar job, binary | Chirikos & Nestel (1984), Anderson and Burkhauser (1986), Chapman & Hariharan (1994) |
| Lagged values of wages and/or health, continuous and binary | Dench & Grossman (2019), Halliday (2017), Haveman et al. (1994) |
| Vocational training, binary | Chirikos & Nestel (1984), Lairson et al. (1984) |
| Black, binary | Chapman & Hariharan (1994), Lee (1982) |
| Occupation, binaries; and 23 occupational categories times average wage for categories within states, continuous | Du & Yagihashi (2017), Ingleby et al. (2021) |
| Marital status, binaries | Chirikos & Nestel (1984) |
| Firm size, binaries | Cai (2009) |
| Measure of intelligence | Grossman (1976) |
| Part-time job, binary | Cai (2009) |
| Non-labor income, binaries | Asher (1984) |
| Index of work organization, continuous | Cottini (2012) |
| Activities of Daily Living index, continuous | Chirikos & Nestel (1984) |
| Work limitation, binary | Wagstaff (1986) |
| Fathers’ and mothers’ education, binaries; and age at birth | Dustmann & Windmeijer (2000) |
| Knowledge of software | Kim & Leigh (2010) |
| State, regional and industry unemployment, continuous | Du & Yagihashi (2017), Wagstaff (1986) |
| State labor force participation, continuous | Du & Yagihashi (2017). |

V. Appendix S-table 4. Authors, samples, methods, findings. (Note: Grossman (1976) and Dustmann & Windemeijer (2000) appear twice: once in the short-run causal and another in the long-run causal.)

|  | Author(s) | Samples | Dependent variables and measure of wages | Methods | Findings |
| --- | --- | --- | --- | --- | --- |
|  | Short-run, causal |  |  |  |  |
| 1 | Asher (1984) | USA. Cross-sectional and longitudinal. 1969 and 1971 waves of the Retirement History Survey (RHS). N = 4890. Only white men. | 2 variables. 1. overall, subjective: equals 1 if health worse than others of same age; = 2 if health same as others; = 3 if health better than others. 2. Indicator of “ability to get around”. Annual earnings. | Least squares. IV. IVs include education, marital status, non-labor income, residence, work experience. Also, Asher uses 1969 values to predict 1971 values. First differences. | Mixed. For 1971 level of health, wages improve health and decrease limitations (p<0.05). For “change in health” from 1969 to 1971, wages significantly harm health and increase limitations (p<0.05). |
| 2 | Cai (2009) | Australia. 2001 and 2003. Cross-sectional and longitudinal. The Household, Income and Labour Dynamics in Australia (HILDA) Survey. N = 2242. Males aged 25-64. | 3 variables. 1.Work limitations. 2. Number diseases doctor says. 3. Overall self-rated health (excellent…poor). Hourly wage = weekly earnings/weekly hours | Ordered probit. 2-stage probit. IV. IVs include work experience and its square, capital city, part-time job, casual job, union membership and firm size. | Cannot reject null hypothesis. No effects of wages on health. |
| 3 | Chirikos & Nestel (1984) | USA. 1976, 1977. Cross-sectional. National Longitudinal Survey of Older Men and Mature Women. Authors use only 1 year from NLS. N = 1609 to 709. Men and women; Blacks and Whites. Ages 45-64. | Presence of work limitation. Hourly wage. IV is “expected” wage calculated even for people with disabilities and not working. | Least squares and IV. IVs include 4 marital status indicators, years of schooling, other training, reside outside south, blue-collar job, age, Inverse Mills Ratio for employment, retired, years of work experience, ADL index. | Wages significantly reduce disability prevalence for all 4 groups (Black men, White men, Black women, White women). Wage effects strongest for Black men. |
| 4 | Cottini (2012) | Europe. 15 countries. 2005. Cross-sectional. European Working Characteristics Survey. Samples vary from N =12,897 to 6030. Ages (likely) 18-64. No gender or race/ethnicity restrictions | 3 health variables; all composites. General health, physical health, mental health. She uses answers to questions involving, for example, skin problems, respiratory difficulties, stomachache, heart disease, stress, sleeping problems, and anxiety. Indicator = 1 if person has low-paid job, 12.2% of sample. Low-pay are earnings below two-thirds of median of earnings. | Probit and least squares. IV. IVs include an index for work organization, minimum wage, and indicator of technology-driven low-pay propensity. | Low pay harms worker health (p<0.01). |
| 5 | Dench & Grossman (2019) | USA. 2007-2011. Longitudinal. National Longitudinal Survey of Youth. N =2497 persons and 11,325 person-years. Majority of sample range from ages 23-27 in 2007 to 27-31 in 2011. No gender or race/ethnicity restrictions. | 5 variables. 1 & 2 Health limits work (yes/no). 3. Annual number injuries and illnesses not treated by doctor (count 1,2,3,4). 4. Same as 3 only treated. 5. Grossman-created variable using overall self-rated (excellent…poor) and scaled to healthy workdays. Mean is 1.73, standard deviation is 0.045. Hourly wage. | First differences. Arellano-Bond method using generalized methods of moments (GMM). IVs include lagged values of health and wages. | No effects of wages on health. “The lack of a causal effect ….may suggest that forces that go in opposite directions in the human capital and compensating wage differential models offset each other.” |
| 6 | Du & Leigh (2015) | USA. Longitudinal. 1999- 2009. Panel Study of Income Dynamics. N = 7029 person-years. Ages 21-65. No gender or race/ethnicity restrictions. | Smoking prevalence; current smoker (0/1). Hourly wage = annual earnings/annual hours. | Least squares. IVs are minimum wages in state and percent unionized in state. Random effects for individuals. | Higher wages reduce smoking prevalence (<0.05), especially for men and people with at most a high school diploma (p<0.01) |
| 7 | Du & Yagihashi (2017) | USA. 2003–2014 American Time Use Survey (ATUS). Repeated cross-section. | Exercise, measured in minutes-per-week | Ordinary least squares (linear regression) but with boot-strapped standard errors to account for the Heckman IV | Higher wages increase time spent exercising. |
| 8 | Dustmann & Windmeijer (2000) | Germany. 1984-1995, 12 years. Longitudinal. N= 2791 to 3324; for person-years N = 7818 to 19,100. | 2 variables. 1. Overall, self-reported measure of health on 11-point scale, from “entirely un-content with health” to “entirely content.” Authors re-scale to 0-1, continuous. Mean = 0.71. 2. Any sporting activity, binary, mean = 0.31. Hourly wage. | Longitudinal, first-differences. Least squares and General Methods of Moments. And IVs that include fathers and mothers education, fathers occupation. Authors distinguish between “transitory” and “permanent” wages. | “Transitory” wages are significantly and negatively correlated with health and sports activity. “Permanent” wages are significantly and positively correlated with health. |
| 9 | Grossman (1976) | USA. Cross-sectional and longitudinal. 1955, 1963, 1969. Two samples. 1. NBER-Thorndike sample of white males, N = 3534. Ages 41-55. 1955 and 1969. 2. 1963 Health Interview Survey, N = 1028. White males. | 3 variables. 1.Grossman-created variable combining data on work loss and overall self-rated health (excellent… poor), (values =1.00, 9.82, 26.41, and 86.68). 2. Overall excellent (yes/no).3. Mortality. Hourly wage. | Least squares and logit. Also IV. IVs include: years of work experience, general intelligence, southern residence, and five measures of city sizes. No IV for mortality analyses. | Wages significantly improve Grossman-created “health stock” variable and overall excellent variable (p<0.01). Insignificant for mortality. |
| 10 | Grossman & Benham (1974) | USA. Cross-sectional. 1963 Health Interview Survey. N = 1049 and 1006 (ages 18-64). White males. | 2 variables. 1.Composite measure using number of symptoms person reported 2. Overall, self-rated: excellent, good, fair, poor. Annual earnings/weeks worked = weekly wage | Least squares. IV. IVs include work experience squared, union member, southern residence, residence in largest SMSAs. | No effects of wages on health. Insignificant |
| 11 | Halliday (2017) | USA. Longitudinal. 1983-1992. Panel Study of Income Dynamics. N = 916 to 3058. Ages 25-60. No race or gender restrictions. | Excellent, very good, good = 1 and fair or poor = 0. But also, change in health using measures excellent = 1, very good = 2, etc, poor = 5. Labor income. | First differences and IVs for lagged values of labor income. | Earnings growth leads to better health. |
| 12 | Haveman, Wolfe, Kreider & Stone (1994) | USA. Longitudinal. 1976-1983. Panel Study of Income Dynamics (PSID). 613 white males, 4640 person-years. Age at least 21 in 1976 and < 65 throughout. | Two work limitations variables measured as 1,2,3 with 3 being worst. Hourly wage = annual earnings/annual hours. | Least squares. IV. IVs include work experience, work experience squared, southern residence, and health status lagged one year. | Wages improve both measures of health, i.e. negatively correlated with limitations (p <0.01 and < 0.05). |
| 13 | Kim & Leigh (2010) | USA. Longitudinal. 2003, 2005, 2007. Panel Study of Income Dynamics. N= 6312 person-years. Household heads age 20-65. | 2 variables. Obesity prevalence (0/1) and BMI. Hourly wage = annual earnings/annual hours. | Least squares. IV. IVs include software knowledge and minimum wages. Longitudinal data with random effects for individuals. | Higher wages reduced BMI and obesity prevalence (p<0.05) |
| 14 | Lairson, Lorimor & Slater (1984) | USA. Cross-sectional (since only used data from 1966). 1966. National Longitudinal Survey, Mature Men. Beginning 1966. Ages 45-59. N = 1471 White men; N = 569 Black men | Variation on Grossman’s Health Stock measure using overall, self-rated measures (excellent…poor) combined with medical spending per-person. Authors create 4 values. Hourly wage. | Least squares. IV. IVs include education, years of work experience, whether received technical training, southern residence, 6 dummies for city sizes. | In all 4 regressions, wages are positively correlated with better health. In 3 of 4, the correlations are significant at the 0.05 level. |
| 15 | Lee (1982) | USA. Cross-sectional. 1966. National Longitudinal Survey of Men. Ages 45-59. N= 2976. | 1.Overall, self-reported health: excellent…poor. 2. health limits work (yes/no). Hourly wage. | Probit and ordered probit. IVs include work experience, SMSA residence, southern residence, Black. | Wages improve health for both overall and work-limitation measures. P<0.05 and <0.01 in all regressions |
| 16 | Leigh & Chakalov (2023) | USA. Longitudinal. 1999-2005. Panel Study of Income Dynamics. Employees, age 18-70. N = 8446 – 37,117 person-years. | Current smoker, binary. Hourly wage. | IV. Union membership and covered by union contract. | Wages reduce smoking |
| 17 | Rodriguez, Targa & Belzer (2006) | USA. Longitudinal. 1995-1998. N= 2368 truck drivers who received pay increases during a period of up to 25 months. 16% of drivers left sample. | Vehicle crashes. Not necessarily crash-with-injury, but crashes by themselves may be viewed as health behaviors. | Pre-post, longitudinal, Event study. Pay rate, cents-per-mile. From 28 cents to 38 cents per mile. Pay = cents-per-mile. | Crash incidence drops by >50% and significant. |
| 18 | Sedigh, Devin, Grenier & Armstrong (2017) | Canada. Repeated cross-sections.2005 and 2010. Canadian General Social Surveys (GSS) − Time Use, for 2005 and 2010. 6455 (4443 non-insomniacs and 2012 insomniacs) in 2005 and of 4668 (3160 non-insomniacs and 1508 insomniacs) in 2010 | Minutes of sleep among people with insomnia. Hourly wage. | Least squares. IV. IVs are union status, education, and industry dummies. | Higher wages result in fewer minutes of sleep among insomniacs. Significant. |
| 19 | Sundberg (1998) | Sweden. Cross-sectional. 1991. Swedish Level of Living Survey. Ages 18-76 years. N = 3322 (1660 women and 1662 men). | Overall ill health. Log-normal. “Bad” = 11 (2% of sample); “in between” = 4 (13% of sample); “good” = 0.8 (84% of sample). Hourly wage. | Least squares. IVs include work experience and work experience squared. Hourly wage. | For women and men separately, wages negatively and significantly correlate with ill-health (p< 0.05). For genders combined, negative correlation but insignificant. |
| 20 | Wagstaff (1986) | Denmark. Cross-sectional. 1976. Danish Welfare Survey (DWS). Ages 20-70. All persons in the labor force. N = 2243. | 4 variables. Single composite measure using indicators such as “often have insomnia”, “pain in back/loin”, “respiratory problems”, “prolonged colds”, “ability to climb stairs”, 19 indicators in all. Also number of doctor visits, hospital stays, and number of complaints about medicine use. Hourly wage. | Maximum likelihood. And a version of IV. IVs include regional unemployment, industry unemployment, and a measure of work limitations. Hourly wage. | Wages improve composite measure of health (p<0.01) but mixed and mostly insignificant effects on medicine, doctor visits and hospital stays. |
| 21 | Woo & Shook (2023) | US. Cross-sectional. Authors collected own data from a hospital. N = 166. | 2 “hardship” variables that measure ability to pay for medical care and food insecurity. Also “financial insecurity”; or anxiety over finances. Hourly wage. | Event study, before and after workers received a raise. Hospital and union negotiated a raise for July 2016. | Higher wages reduce both measures of hardship (p< 0.05 and < 0.01) |
| 22 | Xu (2013) | USA. Multi-year Cross-sections. Overall, 1976-2005. Current Population Survey, CPS (1976-2005), the Behavioral Risk Factor Surveillance System, BRFSS (1984-2005) and the National Health Interview Survey NHIS (1976-2001). N = 460,841 to 146,539. | 9 variables. In BRFFS: Whether smoker (0/1); whether smokes >10 and > 20 per day (0/1); any alcohol use in last 30 days (0/1), any binge drinking (0/1), heavy drinker (0/1), exercise (0/1). From NHIS, any doctor visits in last 12 months (0/1), >1 doctor visits in last 12 months (0/1). Hourly earnings = Annual earnings/annual hours. | 2-sample, 2-stage IV. IV wage created with CPS and then coefficients used on covariates from BRFSS and NHIS. IVs include 8 industry mix variables (eg. % of state employment in manufacturing) and 36 interaction terms (e.g. state unemployment x age category x % in manufacturing). 44 IVs. | Increasing wages caused by economic expansions results in higher smoking prevalence (p<0.01) and >10 and > 20 cigarettes (p<0.05 and <0.10). No effects on binge or heavy drinking or exercise or physician visits. |
|  | II. Short-run, non-causal with longitudinal data |  |  |  |  |
| 1 | Henseke (2018) | Europe. Longitudinal. Individuals. 2004-2013. Survey of Ageing, Health and Retirement in Europe (SHARE). Wave 2 (2006/07) through wave 5 (2012/13). Employed people aged 50–65 years in 15 countries. N = 23,116 person-years. | Self-reported heart attack-stroke-cancer (0/1), cardiovascular-risk-factors (0,1,2,3), musculoskeletal-disorders (0,1,2,…11), mental health (0,1,2,…12), functional disabilities (0/1), overall self-assessed health ( excellent=1,….5=poor). “Monthly pay.” | Least squares, longitudinal, random effects. IV for whether employed (Heckman’s inverse mills ratio) but no IV for wage. | Mixed, mostly insignificant. Higher wages sometimes correlate with better musculoskeletal and mental health but also sometimes correlate with more cardiovascular risk factors e.g. hypertension; no robust correlations. |
| 2 | Leigh & Du (2012) | USA. Longitudinal. 1999-2005. Panel Study of Income Dynamics. N= 17,295 person-years and N =5651 people. Ages 25-65. | 2 variables. “Has a doctor ever told you that you have hypertension?” yes/no. 2. Also, “time to hypertension”. Hourly wage. | Longitudinal logistic with random effects for individuals. Cox proportional model. No IV. Prospective since no person in first year had hypertension. | Wages reduce hypertension incidence, significant (p<0.05); this was especially true for women and people age 25-44 (p<0.01) |
| 3 | Lindeboom & Kerkhofs (2009) | Netherlands. Longitudinal. 1993,1995. Leiden University Center for Research on Retirement and Aging (CERRA) panel survey. Men only, ages 43-63 in 1993. N = 3038 | Composite measure. 57 items of the Hopkins Symptoms Checklist (HSCL). Total health score, integer values between 0 (best health) and 171(worst health). Annual income. | Longitudinal with random effects for individuals. No IV for wage but there is an IV for health to remove bias from “error-ridden” measure of health. | “We also find that higher incomes [from work] are associated with better health.” (p<0.10) |
| 4 | Nocera & Zweifel (1998) | Switzerland. Longitudinal. Various years 1981 to 1993. Data from large Swiss insurance company. Two samples. N = 1908 and N = 2544. | 2 variables 1. subjective health status marked on a visual analog scale ranging from 0 (=very bad) to 4 (=excellent). 2. Annual medical spending by sick fund. Hourly wage = monthly earnings/monthly hours. | Longitudinal data with random effects for individuals. No IV. | Mixed. In one sample (N=1908) wages are positively and significantly correlated with good health; in second sample (N= 2544), wages are insignificant. For the medical care use regressions, wages are never significant. |
|  | III. Long-run causal with longitudinal data |  |  |  |  |
| 1 | Ahammer, Horvath, & Winter-Ebmer (2017) | Austria. Longitudinal. 1992 to 2012. Social Security, tax files, and death records; workers ages 40-60 years in 2002. N =653,803 men and 510,653 women | Mortality over 10 years. Total labor income received in 2002. | IVs are fixed effects for firms. | Cannot reject null hypothesis of no effects of annual 2002 wages on mortality over 10-year period. Insignificant |
| 2 | Anderson and Burkhauser (1986) | USA. Longitudinal. 1969-1976. Retirement History Survey from the Social Security Administration. N = 5836 to 6463 | 2 measures. 1. Health limits work (yes/no). 2. Mortality; died before 1979. Hourly wage. | Logistic with IVs. IVs include white-collar job, 7 measures of education (e.g. = 12 years of schooling, = 16 years, >16 years), married, Black, age. | Higher wages reduce work limitations and mortality (p<0.01). |
| 3 | Chapman & Hariharan (1994) | USA. Longitudinal. 1969-1976. Retirement History Survey from the Social Security Administration. N = 4878. | Mortality; died before 1979. Hourly wage. | Weibull survivor analysis with IVs. IVs include white-collar job, 7 measures of education (e.g. = 12 years of schooling, = 16 years, >16 years), Black. | Higher wages reduce mortality (p<0.01). |
| 4 | Dustmann & Windmeijer (2000) | Germany. Longitudinal. 1984-1995. 12 years. For individuals, N= 2791 to 3324 ; for person-years N = 7818 to 19,100. | 2 variables. 1. Overall, self-reported measure of health on 11-point scale, from “entirely un-content with health” to “entirely content.” Authors re-scale to 0-1. Mean = 0.71. 2. Any sporting activity, binary, mean = 0.31. Hourly wage. Authors draw distinction between “transitory” and “permanent” wages. | Longitudinal, first-differences. Least squares and General Methods of Moments. And IV. IVs include fathers’ and mothers’ education, fathers’ and mothers’ age at birth, fathers’ occupation. | “Permanent” wages are positively and significantly correlated with health and sports. “Transitory” wages are negatively and significantly correlated with health and sports. |
| 5 | Grossman (1976) | USA. Longitudinal. 1955-1969. NBER-Thorndike sample of white males, N = 3534. | Morality. Hourly wage. | Least squares and logit. Also, IV. IVs include years of work experience, general intelligence, southern residence, and five measures of city sizes. | Effect on mortality was insignificant. |
|  | IV. Long-run, non-causal with longitudinal data |  |  |  |  |
|  | Christia (2009) | USA. Longitudinal. 1951-2003. Survey of Income and Program Participation (SIPP), Social Security records and federal taxable earnings. N = “130,000 individuals aged 35–75… approximately 1.2 million person-years.” | Mortality over 3 to 21 years. Long-term aggregate earnings from jobs, 1951 to 2003. | Logistic regression with odds ratios. | Higher long-term earnings associated with lower mortality rates. |
| 2. | Duggan, Gillingham, & Greenlees (2008) | USA. Longitudinal. 1937-2002. Social Security records. Administration. N = “nearly 550,000”. | Mortality. “….nearly half of retired-worker beneficiaries in our sample have died.” Lifetime earnings, ages 35 to 60. | Survival curves and logistic regression. | Higher lifetime earnings predict lower chance of death. |
| 3. | Duleep (1986) | USA. Longitudinal. 1968-1978. Current Population Survey, Internal Revenue Service and Social Security records. N = 9618 with 762 deaths. For disability status N = 9,618 with 144 disabled. | Mortality and newly disabled (disability incidence). Earnings in year prior to death or survival. Also, cumulative earnings from 1968-1972 for mortality. For disability status, 1966 annual earnings and average annual earnings 1964-1966. | Ordinary Least Squares and survival curves. | Low (≤ $2999 in 1972 dollars) annual earnings and low average annual earnings associated with higher mortality (p<0.01). No consistent associations for earnings above $3000. For low to moderate annual earnings ($0-$8,999 in 1972), both annual and average annual earnings associated with “becoming disabled” (p<0.01). |
| 4 | Fletcher et al. (2010) | USA. Longitudinal. 1984-1999. Panel Study of Income Dynamics. N = 4651 to 41,178 person-years depending on the sample and analysis. No restrictions on race or gender. People need not be employed every year. | Self-reported health (excellent,…, poor),  Wage. 5-year cumulative labor earnings. | Ordered Probit with random effects. | Cumulative labor earnings were positively and significantly associated with good health. S-table 4, men, p<0.01 in 6 of 6 regressions. S-table 6, women, p<0.01 and <0.05 in 4 of 5 regressions. |
| 5 | Ingleby et al. (2021) | UK. Longitudinal. Office of National Statistics Longitudinal Study. 2001-2012. 174,931 men and 1962 deaths; 188,844 women and 2063 deaths. No race restrictions. | Mortality. IV for wage including gender, age, and standard occupation code as instruments. 5 wage quintiles. But authors did not know this was an IV and did not make corrections or standard errors. | Poisson regression | Highest wage quintile had lower mortality than lowest quintile. |
| 5 | Kezios, Zhang, Kim, Lu, Glymour, Elfassey, Hazzouri (2022) | USA. Longitudinal. Individuals. 1992-2016. Health and Retirement Survey. Age 50+. N= 2879. Subjects reported wages 2 or more times during 12-year periods. | Composite memory score measured from -1.69 to 2.00 “standard units.” Key independent variable is low-wage with < 2/3rds of federal median wage. Person sustained this wage throughout. | Linear mixed-effects models with random intercepts | “… sustained exposure to low-wage employment in middle age was associated with accelerated memory decline.” (p<0.05) |
| 6 | Kezios , Peiyi , Calonico, Zeki, Hazzouri (2023) | USA. Longitudinal. Individuals. 1992-2018. Health and Retirement Survey. Age 50+. N= 4002. Subjects reported wages 3 or more times during 12-year periods during either 1992-2004 or 1998-2011. | Mortality. Low-wage workers defined as earning less than federal poverty line. | Cox proportional hazards and additive hazards regressions. Controls for measurers of health at baseline. | Low-wage workers more likely to die (277 excess deaths per 10,000; p<0.05) than non-low-wage workers. |
| 7 | Schmitz (2016) | USA. Longitudinal. Individuals. 1992 – 2010. Health and Retirement Survey and 1980 to 2008 Social Security Administration’s Master Earnings File. N = 7394 person-years. Age 50+ in 1992. Full-time male workers ages 50-65. No upper restriction. People followed until they drop out of sample. | Overall, Self-assessed health. Excellent or very good = 1; good, fair, poor = 0. Also, “(1) doctor diagnosed heart disease, (2) doctor diagnosed hypertension, (3) cognitive functioning (total word recall summary score), and (4) depression score. Key independent variables are earnings in 1980 and average annual growth of earnings from 1980 to 1992 | Covariates include lagged measures of overall health, heart disease, hypertension, cognitive function, and depression. Least squares, Probit, random effects. | Earnings from 1980 not statistically significant in predicting self-assessed health from 1992 to 2010. Earnings growth from 1980 to 1992 is significant and positive predictor of self-assessed health (p< 0.05). Earnings in 1980 is positive and significant (p < 0.01) predictor of good cognitive functioning whereas earnings growth is not. Neither 1980 earnings nor earnings growth predicts hypertension or heart disease or depression. |
| 8 | Wolfson, Rowe, Gentleman & Tomiak (1993) | Canada. Longitudinal. 1979-1989. Canadian and Quebec Pension Plans. N= 545,769 males ≥ age 65. | Mortality over 10 years. Cumulative earnings over 13 years before retirement. | Ordinary Least Squares. | Higher earnings predict longer life expectancy (p<0.01). |

VI. Long form descriptions of studies. Alphabetical by author; no separation by short- or long-run or causal.

S1. Ahammer, Horvath, & Winter-Ebmer (2017). The effect of income on mortality – new evidence for the absence of a causal link. *Journal of the Royal Statistical Society Series A: Statistics in Society.* 2017. 180 (3): 793-816.

Data. Austria. Longitudinal. 1992 to 2012. “… matched employer–employee data from the Austrian social security database linked with administrative tax files and death register records” “…all workers between ages 40 and 60 years employed on April 1st, 2002” N =653,803 men and 510,653 women

Demographics. No restrictions on gender or race/ethnicity. Workers ages 40-60 in 2002.

Health. Our outcome is a binary variable equal to 1 if the person died within 10 years after the cross-section (i.e. until December 31st, 2012).

Wage. “…log(annual gross income) received in 2002 according to tax files. Wages are constructed from yearly incomes; thus, they include all monetary benefits that a person receives from the firm.”

Covariates. “…firm size, tenure, experience, unemployment spells between 1992 and 2002, commuting distance, the number of different jobs at the time of the cross-section and full sets of occupational class, education, industry sector, age, neighbourhood population size and country-of-birth dummies. Moreover, we use mean days of sickness leave per co-worker per year between 1992 and 2002 and mean days of sickness leave following work accidents or occupational diseases per co-worker per year between 2000 and 2002 as measures of workplace security.”

IV ? Yes. IVs are fixed effects for firms. I could not find how many firms or dummies for firms they used. Likely 10s of thousands. Authors provide positive tests for validity and strength.

Method. IV (= two-stage-least-squares).

Findings. No effects of annual 2002 wages on mortality over 10-year period.

Comment. Annual wages include hours and days worked or not worked. No account for time-to-death; simply 0/1 dead over 10 years. Cox would have helped.

S2. Anderson KH, Burkhauser RV. The retirement-health nexus: A new measure of an old puzzle. *Journal of Human Resources.* 1985. 20 (3): 315-330.

Data. USA. Longitudinal. 1969-1979. Retirement History Survey from the Social Security Administration. N = 4878.

Demographics. Men only. No restriction on race/ethnicity. Ages 58-63 in 1969.

Health. 2 measures. 1. Health limits work (yes/no). 2. Mortality; died before 1979.

Wage. “Instrumented” permanent wage using 1969 wage regressed on many instruments. Authors call this “permanent wage.”

Covariates. Measure of wealth, Black, age, married, number of children.

IVs. White-collar job, 7 measures of education (e.g. = 12 years of schooling, = 16 years, >16 years), married, Black, age. No tests for strength or validity.

Method. Logistic with IVs.

Findings. Higher wages reduce work limitations and mortality (p<0.01).

Comment. IVs are lousy. Likely standard errors were not corrected for IV. Men only. Also, death is binary, not time-to-death.

S3. Asher CC. The impact of social support networks on adult health. *Medical Care.* 1984. 22 (4): 349-359.

Data. USA. Longitudinal. 1969 and 1971 waves of the Retirement History Survey (RHS) from the Office of Research and Statistics of the Social Security Administration. “The original sample consisted of 11,153 noninstitutionalized persons aged 58-63 years in 1969, although the sample used here is considerably smaller.” N = 4890. “I ….. estimated the individuals' health in 1971 as a function of the events reported in the 1969 survey.”

Demographics. Ages 58-63 in 1969. All persons employed in 1969. White males only.

Health. 4 measures. 1. Subjective, “HT71= 1 if health in 1971 was worse than that of others the same age; HT71 = 2 if health was the same as that of others the same age; and HT71 = 3 if health was better than that of others the same age.” 2. “LIM1= 1 indicates that the individual's ill health limits his ability to get around.” 3. Change in HT71 and 4. Change in LIM1. (Asher is unclear, but presumably, change in HT71 = HT71 – HT69 and the same for LIM1.)

Wage. Annual labor earnings reported to Social Security Administration. No adjustment for work hours.

IVs ? No. Asher never says which are the IVs (instruments) for any of the 3 variables. Perhaps the instruments are measures of the independent variables (married, work experience etc) in 1969 since her IV is called E71HAT, i.e, predicted values of labor earnings in 1971.

Covariates: Married, widowed, divorced, single, years of schooling, years of work experience, 6 occupations (professional, farmer, manager, clerk, craft, operative), number of doctor and/or hospital visits, dummies for medical expenditures, number of times person moved from 1969 to 1971, dummy for working in 1969 but retired in 1971, index of social support networks ( e.g. number of contacts with friends, with siblings) . Remember, white males only.

Method. 2SLS. 3 equations: health (measured either as health of limitations), retirement, wages. Asher never says which are the IVs (instruments) for any of the 3 variables. We infer those years of work experience is one IV. See the bottom of page 351 that mentions “experience” in earnings equation but notice that variable does not appear in any health equation. Asher’s IV is called E71HAT, i.e, earnings, 1971, predicted values.

Findings. For the level of health (not the change) “Nonlabor income (NONWY/100) is insignificant in both equations, while earned income (E71HAT) is significant in both equations, decreasing the probability of ill-health.” For the change in health, earned income (E71HAT) now increases the probability of health worsening for both HT and LIM, while nonlabor income becomes significant and increases the probability of health improving” for both HT and LIM. Coefficients cannot be interpreted given the nature of the dependent variables.

Comment. Asher uses labor earnings in 1971 to predict the change in health from 1969 to 1971; cart before horse. Would have been better to use labor earnings in 1969 to predict the change since earnings in 1971 can be affected by worsening or improving health from 1969 to 1971. Also, Asher creates an IV for labor earnings in 1971 because apparently many people were not working in 1971 and she did not want to discard those people. She should have just used earnings from 1969 to predict health in 1971.

S4. Cai L. Effects of health on wages of Australian men. *The Economic Record*. 2009. 85 (270): 290-306.

Data. Australia. Cross-sectional and longitudinal. The Household, Income and Labour Dynamics in Australia (HILDA) Survey. N = 2242. “The main results are based on the third wave survey, but the panel nature of the data is explored to check the robustness of the estimates.” The first wave survey was in 2001. Although Cai does not say, likely the third wave is 2003.

Demographics. males aged 25-64. Excluded self-employed. Employed workers only.

Health outcomes. “…. whether they had any long-term health condition, impairment or disability that restricted everyday activities and had lasted or was likely to last for six months or more.” “…. individuals were asked whether they were told by a doctor or nurse that they had the following health conditions: arthritis, asthma, cancer, bronchitis, diabetes, coronary, hypertension and circulatory conditions.” “…the Short Form 36 (SF-36) health status questions ….. ‘Would you describe your health as excellent, very good, good, fair, or poor?” The health limitations data are used only to create IVs for the health status variable (excellent…poor). That is, health status is regressed on the limitations variables and the predicted health status variable is used, but only in some regressions. For the key (Cai S-tables 4 and 5) wage-effects-on-health regressions, health is measured as either excellent… poor or IV versions of excellent…poor.

Wages. Weekly earnings/weekly hours.

Methods. Ordered probit, 2-stage probit, 2-stage least squares, Full Information Maximum Likelihood and two-stage FIML. All 2-stage regressions are versions of IV.

Covariates. “….variables included in both the wage and health equations are marital status, education (four dummies), indigenous status, country of birth (three dummies) and state of residence (six state dummies).

IVs. Work experience and its square, capital city, part-time job, casual job, the interaction between casual and part-time job, union membership status and firm size (six dummies). Tests for strong and valid IVs ? Yes; Hansen J statistics. Cai finds IVs are valid.

Findings. Cai’s S-table 4. Wages are insignificant in predicting health in ordered probit, 2-stage probit, and FIML. “The result here just shows that current wages have no significant effects on current health, but it does not imply that current wages have no effects on future health.” In five alternative specifications (Cai’s S-table 5), wages continued to be insignificant.

Comments. “The reverse effect of wages on health arises according to the well-known health production model of Grossman (1972), where it is argued that health capital can be maintained and improved through investments that depend on resources available, including both economic resources and time. Higher wages imply that more economic input into health production is possible, suggesting a positive effect of wages on health. In contrast, an increase in the return to health increases the opportunity cost of health investments, which leads to individuals being involved in market activity more heavily and less time input into health production (Grossman & Benham, 1974). This line of argument suggests a negative effect of wages on health. Therefore, the direction of the simultaneity bias is ambiguous in theory and can only be determined empirically.”

S5. Chapman KS & Hariharan G. Controlling for causality in the link from income to mortality. *Journal of Risk and Uncertainty.* 1994. 8: 85-93.

Data. USA. Longitudinal. 1969-1979. Retirement History Survey from the Social Security Administration. N = 5836 to 6463 depending on the sample/analysis.

Demographics. Men only. No restriction on race/ethnicity. Ages 58-63 in 1969

Health. Death from 1970 to 1979.

Wage. “Instrumented” permanent wage using 1969 wage regressed on many instruments. Authors call this “permanent wage.”

Covariates. Social Security wealth (present value of future Social Security benefits), household wealth in 1969, married, age, self-rated health in 1969, number of respondent’s parents still alive, dollar value of medical bills.

IVs. White-collar job, 7 measures of education (e.g. = 12 years of schooling, = 16 years, >16 years), married, Black, age. No tests for strength or validity. (Same as Anderson & Burkhauser, on purpose)

Method. Weibull survival analysis.

Findings. Higher wages reduce mortality (p<0.01).

Comment. IVs are lousy. Likely standard errors were not corrected for IV. Men only.

S6. Chirikos TN, Nestel G. Economic determinants and consequences of self-reported disability. *Journal of Health Economics.* 1984. 3: 117-136.

Data. USA. Cross-sectional (they used only 1 year each for men and women in the NLS). National Longitudinal Survey of Older Men and Mature Women. 1976, 1977

Demographics. Ages 45-64. Men and women and Blacks and whites. Hispanics not mentioned. Likely Hispanics included in white. Employed.

Health. Disability status. Answers to 2-part question involving “whether the respondent has any health or physical condition that limits the kind or amount of market work he (she) can do and whether these conditions keep him (her) from holding any job at all.” “….we collapse the two responses and represent disability status as a dichotomous variable assigning a value of one to individuals who report no limitation in the amount or kind of work done, zero otherwise.”

Wage. Never clearly defined. Wagehat is the predicted value of wages using covariates in their appendix S-table 3. “The expected (ln) wage rate proxies the ‘value’ or opportunity cost of time.”

Covariates. An index of Activities of Daily Living (ADL), (i.e. difficulty walking, climbing stairs, stooping, etc) , physically demanding job, walking & standing job, eye/hand coordination job, blue-collar job, all other household income (excluding individuals wages). No covariate for SSDI benefits.

IVs for wage equation. Divorced, separated, widowed, never married, years of schooling, other training, reside outside south, , blue-collar job, age, Inverse Mills Ratio, retired, years of tenure on job, years of work experience, ADL index. Covariates common to both wage and disability-status equations: physically demanding job, walking & standing job, eye/hand coordination job. NO tests conducted for validity or strength of IVs.

Findings. Wages are positively and significantly correlated with NOT reporting a disability. “A ten percent drop in expected wage rates raises the probability of disablement by 1.3 percentage points for white women but about twice that amount for black women and white men. Wage effects are strongest for black men.”

Comment. Their S-table 1 presents regression results explaining disability status. No covariates appear for age, schooling, marital status and so many other covariates common to other studies as well as included in their Appendix S-table 3 explaining wages.

S7. Christia JP. Rising mortality and life expectancy differentials by lifetime earnings in the United States. *Journal of Health Economics.*  2009. 28 : 984-995.

Data. USA. Longitudinal. 1951-2003. Survey of Income and Program Participation (SIPP) 1984, 1993, 1996, and 2001, matched to several files from Social Security Administration (SSA) containing information on earnings, disability, and mortality, 1951-2003. Christia also uses federal taxable earnings. (As we know, earnings measure income from jobs, not capital gains, rents, dividends, interest, government benefits or other passive income). N = “130,000 individuals aged 35–75, for which the mortality window ranges from 3 to 21 years, yielding a total of approximately 1.2 million person-year observations.”

Demographics. No restrictions on gender, race/ethnicity, but ages are 35-75 beginning in 1984 or 1993 or 1996 or 2001.

Health. Mortality.

Wage. Long-term aggregate earnings from jobs, 1951 to 2003; 52- years maximum if person is alive in 2003 (but likely not working since youngest person in sample would be 35 + 52 = 87 years old).

Covariates. Age (35-49, 50-64, 65-75 indicators), gender, white, black, other race, Hispanic, less than high school, high school, some college, college or more, never married, married, separated/divorced, widowed, whether on Social Security Disability.

IVs. No.

How to address reverse causality ? “ …..individuals are classified using average lagged earnings which are computed excluding from the average years immediately preceding when mortality is ascertained. In this way, the problem of reverse causality is at least partially addressed by distancing the measurement of earnings from the measurement of mortality.” “Still, the issue of reverse causality is not solved in this approach as individuals can face shocks that reduce their permanent earnings potential as well as increase their baseline mortality risk.”

Method. Logistic regressions that produce odds ratios for the probability of dying.

Findings. Higher long-term earnings associated with lower mortality rates. “This study also finds a substantial increase in life expectancy differentials by lifetime earnings: the top-to-bottom quintile premium increased 30 percent for men and almost doubled for women.”

Comment. As with some other long-run studies, no attempt was made to remove the effects of unemployment.

S8. Cottini E. Health at work and low pay: A European perspective. *The Manchester School.* 2012. 80 (1): 75-98.

Data. Europe. 15 countries. 2005. Cross-sectional. Individuals. European Working Characteristics Survey. Samples vary for 12,897 to 6030.

Demographics. No gender or race/ethnic exclusions. Individuals must be employed “full time”. But she never defines “full time.” She does consider another variable, “long hours” which reflects > 40 hours per week. Age must be < 65.

Health. Her questionnaire asks: “Does your work affect your health, or not? If yes, “how does it affect your health?”: (1) skin problems; (2) respiratory difficulties; (3) stomachache; (4) heart disease; (5) stress; (6) sleeping problems; (7) anxiety and (8) irritability’. Out of the above responses I construct a set of dummies that take value 1 if the worker mentions the problem and 0 if the problem has not been mentioned. For example, individuals were classified as reporting ‘skin problems’ if they answered that their job affected in some way their health and chose ‘skin problems’ as one of the consequences among a checklist of several options. Using all the specific health variables, I built a composite index of general health (healthgen), obtained summing all the dummies defined above. Then, as a measure of the intensity of the physical health problems, I sum up dummies from (1) to (4)—as described above—and construct a composite index (physicalh). I replicate the same procedure to measure mental health problems (mentalh), summing up dummies from (5) to (8).”

Wage. Indicator (dummy) = 1 if person has low-paid job. 12.2% of sample in low-paid jobs. “low-pay (LP) employment as those workers whose earnings fall below two-thirds of the median of the earnings distribution.”

IV? Yes. “(i) an index of high-performance work organization, (ii) minimum wage regulation implemented at the country level, and (iii) an indicator of technology-driven low-pay propensity.” No tests for strength or validity.

Method. Country-level fixed effects. Probit and Least Squares. Instrumental Variables.

Findings. “Low pay plays a role when interacted with working conditions, suggesting that at the lower end of the wage distribution, workers may experience a trade-off between pay and working conditions.” “Results hold also when I account for the endogeneity of low pay and working conditions, and the IV estimates provide evidence in favour of a causal effect of bad working conditions and low pay on workers’ health at work. Overall the results suggest that working conditions and pay levels are important determinants of health status at the workplace…”

### Comment. Cottini has 2 similar papers but neither allow wages to be endogenous. 1. Is it the way you live or the job you have? Health effects of lifestyle and working conditions. *BE Journal of Econ Analysis and Management.* 2017. 2. Is your job bad for your health? Explaining differences in health at work across gender. *International Jo of Manpower* 2012.

S9. Dench D, Grossman M. Health and the wage rate: Cause, effect, both, or neither? New evidence on an old question. *Health and Labor Markets* (*Research in Labor Economics, Vol. 47*), Emerald Publishing Limited, Leeds, UK. 2019. pp. 1-24. <https://ebookcentral.proquest.com/lib/ucdavis/reader.action?docID=5796936&ppg=16&pq-origsite=primo> Accessed January 30, 2024.

Data: USA. Longitudinal. 1997-2011. National Longitudinal Survey of Youth 1997 (NLSY97). This is a sample of the US population consisting of 8,984 individuals who were aged 12 -16 as of December 31, 1996. Two subsamples make up the NLSY97 cohort. “The cohort was interviewed each year from 1997 to 2011 and every other year starting in 2013. Since the maximum number of health measures are available in the period from 2007 to 2011, we limit our sample to individuals who reported data in all those ﬁve years.” N =2,497 persons and 11,325 person-years.

Demographics: “A majority of the sample range in age from 23 to 27 in 2007 and from 27 to 31 in 2011.” People had completed formal schooling by 2006 (before analysis) and were continuously in the labor force. NLS1997 oversamples Blacks and Hispanics.

Health. Five variables. 1. “An indicator of whether health limits the kind of work a respondent can do”, 2. “….an indicator of whether health limits the amount of work a respondent can do”, 3. “the number of times a respondent was injured or ill in the past year and not treated by a physician or a nurse” , 4. “the number of times a respondent was injured or ill and was treated by a physician or a nurse.” Variables #3 and #4 range from zero to four or more. “We assign a value of four to the top-coded category.” All of these measures are negative correlates of better health. 5. “Self-rated health has ﬁve categories: excellent, very good, good, fair, and poor. We scale this variable using a procedure developed by Grossman (1972, 1976).” Grossman scales excellent…poor to work loss days using the National Health Interview Survey. This variable is monotonically increasing with healthy workdays, i.e. days without work loss. This variable is positively correlated with good health. The mean of the scaled variable is 1.73 with standard deviation of 0.045. Binary (indicator) “limits” variables (#1, #2) have very small means, 0.02.

Wage. “The wage rate itself is the one reported by the respondent on his or her full-time or part-time job. If a respondent has a fulltime and a part-time job, then the wage from the full-time job is used. If he or she has multiple full-time jobs, the simple average of the full-time jobs is used. If a respondent has multiple part-time jobs and no full-time jobs, the simple average of part-time jobs is used.”

Covariates: age, white, Black, Hispanic, other race, female, high school or less, some college, college or more, years of work experience, indicators for year of survey, married, own income other than work-earnings, spouses income, number of kids

IVs. Yearly-lagged levels of health and wages are used for instruments. F- Tests for instruments are strong. They claim instruments are valid via arguments, no tests.

Methods: First differences. They use the Arellano-Bond dynamic panel estimates of the generalized methods of moments (GMM) ﬁrst-differenced for health and wage equations. Years-lagged values on health and wages are viewed as IV variables (instruments). “The estimates in each S-table employ up to three lags of health and up to three lags of the wage as instruments.” They also use only the second lag of health and only the second lag of the wage in some analyses. Under these circumstances, “since the equations are exactly identiﬁed, the Arellano-Bond ﬁrst difference procedure is identical to conventional two-stage least squares.” They do not weight the data to adjust for oversampling of minorities.

Findings: “There is no evidence of a causal relationship running from the wage rate to health.” They find reductions in health lead to increases in wages which they attribute to the effects of theory of compensating wages for dangerous or unhealthy work.

Comment: The Arellano-Bond approach provides better IV variables (instruments) and better overall methods than the three key simultaneous equations studies by Grossman and Benham (1974), Grossman (1976), and Lee (1982). “By implementing dynamic panel data models that allow lagged levels of health and the wage to be specified as instruments for differences of right-hand-side measures of these variables on theoretical grounds, we relax the arbitrary identification assumptions in the studies by Grossman and Benham (1974), Grossman (1976), and Lee (1982).” But the finding that poor health leads to higher wages is shocking; we are not aware of any other health-effects-on-wages study that finds that. Authors attempt to explain this finding by appealing to the presence of compensating wage differentials. Another comment: “Our estimates are limited to continuously employed workers. That raises a standard selection issue because reductions in health could lead not only to effects on wages but also to spells out of the labor force. Ideally, we would want to estimate an econometric model that deals both with the simultaneity issue, as we do, and with the selection issue. One reason we have chosen not to estimate a sample selection model is that this model is not highly credible without theoretically based exclusion restrictions. A second reason is that the three previous papers (Grossman, 1976; Grossman & Benham, 1974; Lee, 1982) that estimated simultaneous-equations health-wage models limited their estimates to workers in the labor force.” AND “While we find some evidence that a reduction in health leads to an increase in the wage, that evidence is limited to continuously employed workers. It might be weakened or even reversed if we did not limit the sample to that group. For example, a worker who is forced to leave the labor force due to poor health and then returns a year or two later may have a lower wage than an otherwise identical continuously employed worker due to a loss of experience and on-the-job investment.” ALSO “A good deal of caution is required in interpreting our findings and in extrapolating them to the population at large. Clearly, the results pertain to a selected sample. The panel members were at the early stages of their working careers during the period at issue and were continuously employed during the Great Recession. The realization that many of their colleagues were unemployed or could not find jobs may have caused them to increase their work effort, which resulted in reductions in health but led to subsequent wage increases. This may be one subtle factor in the findings in some studies that positive correlates of health rise in a recession (e.g., Cutler, Huang, & Lleras-Muney, 2016; Ruhm, 2000, 2003, 2005). “

S10. Du, J. and Yagihashi, T., 2017. Health capital investment and time spent on health-related activities. *Review of Economics of the Household*, *15*(4), pp.1215-1248.

Data. USA. 2003–2014 American Time Use Survey (ATUS). Repeated cross-section.

Demographics. Ages 25- 65; not in school, or armed forces, unemployed, those not in the labor force.

Health. Time spent : “(a) exercise; (b) medical + personal care; (c) socializing + relaxation.” And non-religious tv viewing and sleeping. We only included results on exercising. See comments below.

Wage. “Wage rates are measured as per-hour earnings for workers paid hourly.” “For nonhourly workers, we use their weekly earnings divided by their hours worked.” And “…. real wage rate predicted from a Heckman sample selection equation.”

Covariates. “..gender, age, age squared, race, educational attainment, spousal earnings, marital status, number of children per age bracket (0–2 year old, 3–6 year old, and 7–18 year old), urban/rural, and three regional dummies (Northeast, Midwest, or South).” “….price of related market goods (constructed by authors) and average temperature and total precipitation for the month of the interview. The model additionally controls for day of the week indicators (ω), year dummies (μ), state dummies (ϕ), and an indicator for summer months (s).”

IV? Yes. Heckman method. “….state labor-force participation rate, unemployment rate, and the minimum wage.” And 23 broad occupational categories combined “with the average hourly wage for that occupation in each state and year.” Authors tested for strength and validity and found positive results , i.e. instruments were both strong and valid.

Method. Ordinary least squares (linear regression) but with boot-strapped standard errors to account for the Heckman IV.

Findings. Strongest results indicate higher wages lead to more exercise.

Comment. To remain consistent with all other studies we reviewed, we omit results on medical and personal care. All other studies measure medical care with currency. This is the only one that measures that care with time. We also ignore findings on sleep as this sleep variable is not linked to insomnia (see Sedigh et al). It is not medically sound to attribute any health harm or benefit to sleep among people who do not have sleep problems. Finally, we ignore results on socializing and relaxing as well as viewing non-religious TV because these activities are not widely regarded as behaviors that have direct effects on peoples’ health.

S11. Du J, Leigh JP. Effects of wages on smoking decisions of current and past smokers. *Annals of Epidemiology*. 2015. 25: 575-582.

Data. USA. 1999- 2009. Longitudinal. Individuals. Panel Study of Income Dynamics. N = 7029 person-years.

Demographics. Full-time employed persons, ages 21-65. Excluded adult never-smokers. No restrictions on gender or race/ethnicities. “…employees and/or the self-employed working full time defined as 1750+ annual work hours and 49+ weeks per year. “

Health. Smoking prevalence; current smoker (0/1).

Wage. Estimate of wages-per-hour. “Even though our samples are restricted to full time workers, we divide annual earnings by annual hours to obtain wages-per-hour.”

Covariates. Gender, age, white non-Hispanic, Black non-Hispanic, Hispanic, other, married, years of schooling, whether self-employed, 3 of 4 US regions, state cigarette tax, state unemployment rate, tobacco-controlled funding, number of smoke-free laws. Also, “other family income” i.e. spouse’s wages, capital gains, interest, dividends, government benefits.

IV? Yes, minimum wage in state and percent unionized in state. Authors also tested for strength (f-tests) and validity (Sargan-Hansen J test). Both were favorable for these IVs.

Method. Least squares linear regression, probit, IV. Wages are measured in the year before smoking prevalence is measured.

Findings. “We found some evidence that low wages lead to more smoking in the overall sample and substantial evidence for men, persons with high school educations or less (< 13 years of school) and quitters.”

S12. Duggan JE, Gillingham R, Greenlees JS. Mortality and lifetime income: Evidence from U.S. Social Security records. *IMF Staff Papers.* 2008. 55. 566-594.

Data. USA. Longitudinal. 1937-2002. Continuous Work History Sample (CWHS) and the Master Beneficiary Records (MBR) of the Social Security Administration. “… we selected all retired-worker beneficiary records with birth years 1900 to 1942, who claimed benefits at age 62 or later.” “Because our benefit/death information extends through 2004, the oldest observed age in our sample is 104.” N = nearly 550,000 observations.

Demographics. No restrictions on gender or race/ethnicity. Includes self-employed.

Health. Mortality. “….nearly half of retired-worker beneficiaries in our sample have died.”

Wage. “Lifetime earnings are measured for each individual as the sum of real earnings (2005 dollars) over ages 35 to 60.”

Covariates. White, Black, male, female.

IVs. None.

How to address reverse causality ? “…reverse causation, namely from higher mortality rates to lower incomes. Note, however, that the ages used to construct our lifetime income variable are prior to age 61, and therefore are separate from the years we examine in our mortality function estimates. Moreover, we separate out disabled beneficiaries, thereby minimizing the possibility that people with dramatically lower life expectancies have lower measured lifetime incomes.” “It remains a possibility that it is not income per se that makes individuals with higher incomes live longer, that instead there may be an underlying demographic or other factor at work. For example, some people who are intelligent, educated, or diligent enough to take good care of their health may also attain high incomes because of those same traits. Or, some disadvantaged groups may have low incomes and also lower life expectancies because of childhood-nutrition issues. We have two defenses against this criticism, however. First, we are not trying to conclude that high income individuals purchase higher life expectancy, and we accept the possibility that some latent variable leads to the positive relationship we observe. Second, our analysis of the income-mortality nexus for dually entitled females shows that the income of the spouse (the primary earner) has a significant effect on mortality for white females.”

Methods. Survival curves and logistic regression.

Findings. Higher lifetime earnings predict lower chance of death. “Logit models by gender and race confirm a negative relationship. Differences in age of death between low and high levels of lifetime income are on the order of two to three years. Income-related mortality differences between blacks and whites are largest at low-income levels, but gender differences appear to be large and persistent across income levels.”

Findings. No account for unemployment

S13. Duleep HO. Measuring the effect of income on adult mortality using longitudinal administrative record data. *Journal of Human Resources*. 1986. 21 (2): 238-251.

Data. USA. Longitudinal. 1968-1978. Three matched datasets: Current Population Survey, Internal Revenue Service and Social Security Admiration. N = 9618 with 762 deaths. For disability status N = 9,618 with 144 disabled.

Demographics. Men, white, married, ages 35-65 in 1973; in jobs covered by Social Security records.

Health. Mortality and disability status (although she uses CPS disability status in some analyses, for the key ones she uses Social Security Disability status and restriction attention to newly reported disability)

Wage. Earnings in year prior to death or survival. Also, cumulative earnings from 1968-1972 for mortality. For disability status, 1966 annual earnings and average annual earnings 1964-1966.

Covariates. Age, education, (remember sample already restricted to white married men). In some regressions, indicator of work disability status.

IVs. None.

Method. Ordinary least squares.

Findings. Low ($2999 in 1972 dollars) annual earnings and low average annual earnings are associated with higher mortality (p<0.01). No consistent associations for earnings categories above $3000. For low to moderate annual earnings ($0-$2,999; $3,000-$5,999; $6,000-$8,999 in 1972), both annual and average annual earnings are associated with “becoming disabled”, most frequently with p<0.01.

Comment. She attempts to control for reverse causality (poor health causes both low earnings and mortality) by including Social Security Disability and CPS status as a covariate.

S14. Dustmann C, Windmeijer F. Wage and the demand for health – A life cycle analysis. Discussion paper 171. July 2000. <file:///C:/Users/pleigh/Downloads/SSRN-id239794.pdf> Accessed March 18, 2024.

Data. Germany. Longitudinal. 1984-1995. 12 years. Unbalanced sample, allows for attrition out and new entrants in. Number of individuals N= from 2791 to 3324 ; person-years N = from 7818 to 19,100.

Demographics. “Working males”, ages 25-60. No race or ethnic categories.

Health. Self-reported measure of health on 11-point scale, from “entirely un-content with health” to “entirely content.” Authors re-scale to 0-1. Mean = 0.71. Also, whether person engages in any sporting activity on weekly basis. Binary, if any sporting activity, then = 1. Mean = 0.31.

Wage. Hourly wages adjusted for inflation with 1984 as the base year. Defined as monthly gross wages divided by monthly hours. Gross wages can include overtime pay.

Covariates. Age, family income, and years of schooling only (it would appear). Authors “difference out” all time-varying variables such as, presumably, marital status (but marital status can change over 12 years!) .

IVs. Lagged levels of wages. Generalized Methods of Moments. Yes, Sargan test for validity and they accept the null hypothesis.

Method. OLS. First differences because they have longitudinal data.

Findings. “Instantaneous” or “transitory” wages are negatively but insignificantly correlated with “health stock” (i.e. subjective score, 0 to 1) and weekly sports activity (their S-table 2), but significant (their S-table 3). “Permanent” wages are positively correlated with both “health stock” and sports activity but only significant for sports activity (their S-table 4). In their abstract, “We find evidence of negative transitory wage effects, and positive permanent effects.”

Comment. Authors never published in a journal. SSRN paper.

S15. Fletcher JM, Sindelar JL, Yamaguchi S. Cumulative effects of job characteristics on health. *Health Economics.* 2010; 20. 553-570.

Data. USA. 1984-1999. Longitudinal. Individuals. Panel Study of Income Dynamics. N = 4651 to 41,178 person-years depending on the sample and analysis.

Demographics. No restrictions on race or gender. People need not be employed every year.

Health. Self-reported health (excellent, …, poor). They use ordered probit.

Wage. 5-year cumulative labor earnings.

Covariates. age, age squared, years of schooling, self-employment status, marital status, time out of the labor force, self-reported health when young, Spells of unemployment, year dummies, cumulative weekly work hours measures of physical and environmental demands at work.

Causal model? Not exactly. No IV or DiD or discontinuity or fixed effects or propensity scores.

Methods. Ordered Probit with random effects. Separate analyses for gender and white/non-white and young and old (>= 40 years) . “We use a relatively parsimonious set of control variables, including age, a quadratic in age, years of schooling, self-employment status, marital status, time out of the labor force, self-reported health when young, year dummies, and in some specifications cumulative weekly work hours and labor income.”

Findings. Cumulative labor earnings were positively and significantly associated with good health. S-table 4, men, p<0.01 in 6 of 6 regressions. S-table 6, women, p<0.01 and <0.05 in 4 of 5 regressions.

Comment. Not exactly causal since(unmeasured) health can affect cumulative labor income in prior years. But health variables are measured in years after cumulative labor income and Fletcher includes some measures of health when young.

S16. Grossman, M. The correlation between health and schooling. In N. E. Terleckyj (Ed.), Household production and consumption (pp. 147-211). *Studies in income and wealth, Vol. 40, the conference on research in income and wealth.* New York, NY: Columbia University Press for the National Bureau of Economic Research. 1976.

Data. USA. Individuals. 1955, 1963 and 1969. Cross-sectional and longitudinal. First data set was the NBER-Thorndike sample of white males. Second data set was the 1963 health interview survey conducted by the National Opinion Research Center (NORC), University of Chicago. For NBER-Thorndike N = 3534; for NORC, N = 1028.

Demographics. White men only for both NBER-Thorndike and NORC. NBER-Thorndike data were candidates for pilots, navigators, or bombardiers in the Army Air Force in the last half of 1943. Re-interviewed in 1969 and 1971. To be accepted as a candidate, a man had to pass physical and scholastic aptitude exams. The aptitude exam screened out the lower half of applicants. The 1963 NORC-national-health interview survey was more representative of white males in the US than Thorndike-NBER. No descriptive statistics are provided. Ages (in 1969) likely ranged from 45 to 55.

Health outcomes. Three outcomes. The first was a Grossman-created variable combining data on work loss weeks and responses to the overall health question (i.e. excellent, good, fair, poor), labeled H69. One loose interpretation is that H69 represents healthy weeks-of-work, i.e. weeks of work without absence due to ill health. H69 values were 1.00, 9.82, 26.41, and 86.68. The second variable was binary and equals 1if respondent answered that overall health was excellent and equals 0 otherwise (for good, fair, poor). The third was mortality.

Wages. ln(wage rate). The hourly wage rate equals a man’s full-time salary on his current job divided by the product of fifty weeks and the average number of hours per week he worked on his main job in 1968. This measure was used for H69 and the binary “excellent” variable. For mortality, wage was measured in 1955.

Methods. Ordinary least squares for effects of wages on health and two-stage-least-squares for reciprocal effects between wages and health.

Covariates. (Loosely interpreted) healthy weeks in high school (similar to H69 only applied to illness absence in high school), age in 1969, years of father’s schooling, years of mother’s schooling, visual perception, psycho-motor control, mechanical ability, general intelligence, numerical ability, years of wife’s’ schooling, job satisfaction (1 to 5 with 5 = best), actual weight-desired weight, other family earnings.

IVs. Years of work experience, general intelligence, southern residence, and five measures of city sizes in which respondent resided . No tests for either strong or valid IVs.

Findings. For all five OLS and three 2SLS regressions when dependent variables were H69 and “excellent”, wage coefficients were positive and significant at the 0.01 level, 2-tailed tests. For the three mortality logit regressions, wage coefficients were insignificant (as were all other covariates except schooling).

Comment. Highly unlikely that the instruments --- years of work experience, general intelligence, southern residence, and five measures of city sizes in which respondent resided--- were valid. Otherwise, an amazingly rich and unique set of covariates.

S17. Grossman M, Benham L. Health, hours, and wages. In *The Economics of Health and Medical Care: Proceedings of a Conference held by the International Economic Association at Tokyo,* edited by Mark Perlman. John Wiley & Sons, New York, NY. 1974.

Data. USA. Cross-sectional. 1963 Health Interview Survey conducted by the National Opinion Research Center (NORC), University of Chicago. N = 1049 (ages 18+) and 1006 (ages 18-64).

Demographics. White males, 18+ years old, and ages 18-64, completed schooling, did not reside on farms, positive earnings.

Health outcomes. Two measures available in NORC. 1. Number of symptoms person reported from a list of 20 possible symptoms. 2. Overall, self-reported (excellent, good, fair, poor). Authors use principal components analysis to construct a composite, index, health variable, IH, that uses information on symptoms and self-evaluation. IH is a negative measure of health; as IH goes up, health goes down. The IH scale is “essentially arbitrary.” “….the estimated effects of IH on wages…” and the effects of wages on IH, “are fundamentally qualitative in nature rather than quantitative.”

Wages. Annual earnings/weeks worked = weekly wage. No direct measure of the wage rate per hour. Excludes sick leave time.

Covariates. Common covariates between wage and health equations: years of schooling and years of work experience. See “methods” below for other covariates.

IVs. Work experience squared, union member, southern residence, residence in one of 10 largest SMSAs, residence in any other SMSA (not the top 10). No test for strength or validity.

Methods. Ordinary Least Squares and Two-stage Least Squares (IV). Three-equation model for health, work hours, and wages.

Findings. No significant direct or direct+indirect effects of wages on health (S-tables 12.9 and 12.10). Significant positive effects of wages on work hours (the indirect effect).

Comments. 1. Useful to think about the indirect effect of wages on work hours as epidemiological evidence suggests excessive work hours harms health. But, presumably, the reduced form equation that omits work hours as a covariate implicitly accounts for the indirect effect. 2. The authors instruments are likely not valid. 3. To their credit, authors include physicians per capita and health insurance as covariates in the health equation. But could argue that wages affect health insurance or that there is a compensating wage differential between wages and insurance (Craig A Olson, Jo Labor Econ, 2002)

S18. Halliday TJ. Earnings growth and movements in self-reported health. *Review of Income and Wealth*, 2017. 63: 760-776. <https://doi.org/10.1111/roiw.12242>

Data. USA. Longitudinal. 1983-1992. PSID

Demographics. Ages 26-60. No race or gender exclusions.

Health. Self-reported health. 1= excellent….5=poor. Binary = 1 if excellent, very good, or good and = 0 if fair or poor.

Wage. Labor income = income from wages, salaries, business income, bonuses, overtime, commissions, professional practices, income from boarders.

Method. First differences. Change in health regressed on change in labor income.

IVs. Lagged measures of labor income.

Findings. Earnings *growth* leads to better health for both married men and women.

S19. Haveman R, Wolfe B, Kreider B, Stone M. Market work, wages and men's health*. Journal of Health Economics.* 1994. 13:163-182.

Data. USA. Individuals. Longitudinal. 1976-1983. Panel Study of Income Dynamics (PSID). 613 white males, 4640 person-years, 3-digit occupational data from the Dictionary of Occupational Titles. Pollution data by county.

Demographics. 613 white males, household heads, at least 21 years old with histories of significant labor force attachment. 4640 person-years, 1976-1983. They look at only white males to allow comparisons to Grossman’s and Lee’s papers.

Health outcomes. Self-reported answers to two questions. 1. “Do you have a physical or nervous condition that limits the type of work or the amount of work you can do?” If the man answers yes, he is asked “Does the condition limit your work a lot, somewhat, or just a little?” Haveman et al. coded this as 0 = no condition, 1 = just a little, 2 = somewhat, 3 = a lot.

Wages. Measured as the log of annual earnings/annual workhours in 1976 dollars.

Method. 3-equation, simultaneous model. Wages are presumed to affect workhours that, in turn, affect health. Neither actual wages nor an IV version of wages enters the equation for health in S-table 1. But wages (or their IV equivalent) are allowed directly into their S-table 2. Haveman et al. use Generalized Methods of Moments, least squares, and a version of IV.

IVs. Unique IV variables (instruments) in the wage equation are years of work experience, work experience squared, southern residence, and health status lagged one year. No IV tests for strength or validity.

Covariates. Age, years of schooling, years of work experience, marital status, strength used on the job, hazards encountered on the job, wife’s schooling, whether self-employed, number of children, residence in south, northeast, Midwest or west, wife’s age, mother's years of schooling, father’s years of schooling, age of youngest child, level of sulfur dioxide in county, county unemployment rate, indicator of respondents ambition in 1972.

Findings. “Finally, like Wagstaff, we find wages to be positively and significantly associated with good health.” (S-table 2). In three regressions, one is significant at the 0.01 level, second is significant at the 0.05 level and third is not significant but has the same sign.

Comment. Haveman et al. (1994) extend Lee's model by adding an equation for endogenously determined hours of work. Unique IV variables (instruments) are difficult to justify. For example, self-employment is assumed to affect hours but not wages, while divorce is assumed to affect health status but not hours or wages and southern residence is assumed to affect wages but not health.

S20. Henseke G. Good jobs, good pay, better health? The effects of job quality on health among older European worker*. European Journal of Health Economics.*  2018. 19:59–73

Data. Europe. 2006-2013. Longitudinal. The Survey of Ageing, Health and Retirement in Europe (SHARE). Wave 2 (2006/07) through wave 5 (2012/13). “…unbalanced panel of employed people aged 50–65 years in 15 countries…” N = 23,116 person-years.

Demographics. No gender, race restrictions. Only ages 50-65.

Health. AHC (heart attack, stroke or cancer) (0/1), CVR cardiovascular risk factors (0,1,2,3), MSD musculoskeletal disorders (0,1,2,…11), MH mental health(0,1,2,…12), FD functional disabilities (ADL and IADL) (0/1), SAH self-assessed health ( 1= excellent,….5=poor).

Wage. Monthly pay. No control for work hours.

IV ? No, not for wages, but yes for any employment. Author uses Heckman inverse mills for any employment.

Covariates. “….age, sex, educational attainment, marital status and country of birth, cognitive abilities, past smoking behaviour, home ownership, socio-economic circumstances and healthiness during childhood.” Random (not fixed) effects for individuals.

Method. Logit, ordered logit, Poisson. Random effects for individuals

Findings. Higher wages most often correlate with better health (p<0.5 and <0.01). But several insignificant findings. But several insignificant findings and positive effects appear “transitional” not long lasting. “…..evidence for genuine protective effects of better jobs on musculoskeletal disorders, mental health and general health. The effect could contribute to a substantial number of avoidable disorders among older workers, despite relatively modest effect sizes at the level of individuals. Mental health, in particular, responds to changes in job quality. Selection bias such as the healthy worker effect does not alter the results.”

Comment. Not causal for wages, but author does Heckman mills ratio (IV) for whether employed.

S21.Ingleby FC, Woods LM, Atherton IM, Baker M, Elliss-Brookes L, Belot A. Describing socio-economic variation in life expectancy according to an individual's education, occupation and wage in England and Wales: An analysis of the ONS Longitudinal Study. *SSM Popul Health*. 2021 May 8;14:100815. doi: 10.1016/j.ssmph.2021.100815. PMID: 34027013; PMCID: PMC8131985

Data. UK. ONS Longitudinal Study. Longitudinal. Office of National Statistics Longitudinal Study. 2001-2012. 174,931 men and 1962 deaths; 188,844 women and 2063 deaths. No race restrictions.

Data. No race or gender restrictions.

Health. Mortality

Wage. Quintiles

Covariates. Age, gender.

Method. IV for wage including gender, age, and standard occupation code as instruments. 5 wage quintiles. But authors did not know this was an IV and did not make corrections or standard errors. Poisson regression.

Findings. Lowest wage quintile had highest mortality rate.

S22. Kezios KL, Zhang A, Kim S, Lu P, Glymour MM, Elfassey T, Hazzouri AZI. Association of low hourly wages in middle age with faster memory decline in older age: Evidence from the Health and Retirement Study. *American Journal of Epidemiology.* 2022. 191 (12): 2051–2062

Data. USA. Longitudinal. Individuals. Health and Retirement Survey. HRS participants born 1936-1941, subjects work for pay during 1992-2004 with data from at least 2 years. N = 2789.

Demographics. No gender or race/ethnic restrictions.

Health. Composite memory score measured from -1.69 to 2.00 “standard units.”

Wage. Binary. Low-wage is wage that is < 2/3rds of federal median hourly wage. “Cumulative exposure to low wages across the study visits from 1992 to 2004 was then classified into 3 categories—“never,” “intermittent,” and “sustained” low-wage exposure—based on all years in which exposure information was contributed. All participants contributed, minimally, 2 time points of wage information and could contribute up to 7 time points.” “On average, participants contributed 4.7 time points of wage information, with 72% of the sample reporting wage information at 4 or more study visits.”

Covariates. “Baseline covariates reported in 1992 included: age, sex, education , race/ethnicity, birth in a southern state, highest level of parental education, working in a low-skilled occupation (low vs. high), and household wealth. An individual’s occupation was defined as highly skilled if it belonged to the highest skill-level category (level 4) according to the International Labour Organization International Standard Classification of Occupations (ISCO-08), and lower-skilled otherwise (levels 1–3) (19, 45). Household wealth (including secondary residence) was assessed in 2016 dollars, …..and equivalized on household size by dividing each participant’s wealth by the square root of the number of household member.”

Method. Linear mixed-effects models with random intercepts. “Time (years) since the beginning of cognitive follow-up (2004) was used as the time scale. The primary coefficients of interest were: 1) the main effect for time (i.e., the slope of the memory trajectory for “never” low-wage earners) and 2) the interaction between time and low-wage exposure history (i.e., the difference in the slopes of the memory trajectories for “intermittent” or “sustained” low-wage earning vs. “never” low-wage earning).”

Findings. “Overall, we observed that sustained exposure to low-wage employment in middle age was associated with accelerated memory decline.” “The confounder-adjusted annual rate of memory decline among “never” low-wage earners was −0.12 standard units (95% confidence interval: −0.13, −0.10). Compared with this, memory decline among workers with sustained earning of low midlife wages was significantly faster (βtime×sustained = −0.014, 95% confidence interval: −0.02, −0.01), corresponding to an annual rate of −0.13 standard units for this group.”

Comment. Do Kezios et al confuse unemployment with low wages? Probably not. “All HRS participants were asked to report their hourly wage using the question, “What is your hourly wage rate for regular work time?”….. A person was identified as a low-wage earner if their hourly wage was lower than two-thirds of the federal median wage for the corresponding year.” So these people must be employed at the time of the survey. On the other hand, low-wage people are more likely to experience unemployment, perhaps after the interview and before the next.

S23. Kezios KL, Peiyi L, Calonico S, Zeki A & Hazzouri A. History of Low Hourly Wage and All-Cause Mortality Among Middle-aged Workers. *JAMA*. 2023. 329 (7). 561-573.

Data. USA. Longitudinal. Individuals. “4002 US participants, aged 50 years or older, from 2 sub cohorts of the Health and Retirement Study (1992-2018) who worked for pay and reported earning hourly wages at 3 or more time points during a 12-year period during their midlife (1992-2004 or 1998-2010). Outcome follow-up occurred from the end of the respective exposure periods until 2018.”

Demographics. No race or gender restrictions. Ages 50+. 4002 workers (age range, 50-57 years at the beginning of exposure period and 61-69 years at the end; 1854 female [46%]).

Health. Mortality

Wage. Low-wage—less than the hourly wage for full-time, full-year work at the federal poverty line—earning history was categorized as never earning a low wage, intermittently earning a low wage, and sustained earning a low wage.

Causal model. None.

Methods. “Cox proportional hazards regression models and Aalen additive hazards regression models, respectively, to estimate the effect of low-wage history on all-cause mortality, using person-years as the underlying time scale.”

Covariates. “Baseline covariates were from the 1992 or 1998 survey waves… and included: sex, educational attainment, racial and ethnic group membership, birth in Southern states, 5 marital status, highest level of parental education, household wealth, household income, body mass index (BMI), smoking status (ever vs never), drinking status (≥3 vs < 3 drinks per day), an index of physician-diagnosed health problems (range, 0-8, a higher number indicates more health problems), self-reported health (fair or poor vs good, very good, or excellent), and elevated depressive symptoms….. work-limiting disability, ….whether the participant ever reported receiving income assistance such as welfare or food stamps, …..how often the participant received employer-provided health insurance , and whether participants ever worked in low-skilled occupations (vs never) over the exposure period using definitions of occupational skill levels from the International Labour Organization International Standard Classification of Occupation (ISCO-08). Based on prior research, level 4 was considered high-skill and levels 1-3, low-skill.”

Methods. “Cox proportional hazards and additive hazards regression models sequentially adjusted for sociodemographics, and economic and health covariates were used to estimate associations between low-wage history and all-cause mortality. We examined interaction with sex or employment stability on multiplicative and additive scales.”

Findings. Low-wage workers more likely to die (277 excess deaths per 10,000; p<0.05) than non-low-wage workers. “Sustained low-wage earning may be associated with elevated mortality risk and excess deaths, especially when experienced alongside unsS-table employment. If causal, our findings suggest that social and economic policies that improve the financial standing of low-wage workers (eg, minimum wage laws) could improve mortality outcomes.”

Comment. Poor health could result in low wages, reverse causality. But authors control for numerous measures of health at baseline. Unlikely authors confuse low wages with unemployment since to be included in the sample, participants must report a wage. However, after the interview and before the next, they might be unemployed. But this is true for all annual surveys of wages.

S24. Kim D-H , Leigh JP. Estimating effects of wages on obesity. *Journal of Occupational and Environmental Medicine.* 2010. 52 (5): 494-500.

Data. USA. Three years: 2003, 2005, 2007. Individuals. Longitudinal. Panel Study of Income Dynamics. N= 6312 person-years.

Demographics. Heads of households. Working full time, no self-employed a between and including ages 20 to 65 years. Excluded full-time students, retirees, permanently disabled persons, self-employed, and non-heads of households. Balanced design, people must report data all 3 years.

Health. BMI and obesity

Wage. Log of hourly wage rate in interview year.

Covariates. Age, age-square, male, white, married, high school graduate, any health insurance, smoker, 3 regions and 2 years as indicator variables

IV ? yes. Instruments are the respondents’ familiarity with computer software and state-by-state minimum wages.

Method. Least squares. IV. Random effects for individuals.

Findings. Higher wages reduce BMI and prevalence of obesity (p<0.05)

S25. Lairson D, Lorimor R, Slater C. Estimates of the demand for health: Males in the pre-retirement years. *Social Science and Medicine*. 1984. 19 (7): 741-747.

Data. USA. Cross-sectional (since only used data from 1966). 1966. National Longitudinal Survey, Mature Men. Beginning 1966. Ages 45-59. Oversamples Blacks. “The sample of 5020 men originally interviewed in 1966 included 3518 white men, 1420 black men and 82 men of other races.” Authors subset: 1471 white respondents and 569 black respondents, married, wage or salary workers in their current or last jobs.

Demographics. Men. Black, white, other race.

Health. Authors guided by Grossman’s measure. “….we averaged medical care expenditures from the 1963 and 1970 Center for Health Administration Studies surveys for black and white males in the 45-59 age group. These figures were used to construct an index of health stock with Hp = 1 for persons who perceived their health status to be poor. To obtain the health stock values, let Mp, Mf, Mg and Me be average expenditures by people in poor, fair, good and excellent health. Then Hf = Mp/Mf, Hg = Mp/Mg, He = Mp/Me determines the health index for persons reporting, fair, good and excellent health.” So authors create 4 values.

Wage. Hourly wage rate.

Covariates. Job satisfaction (1=likes….4=dislikes), family size, family assets, age, years of schooling for man, years of schooling for wife

Method. OLS and 2SLS.

IVs. Yes. For wages: years of work experience, whether received technical training, southern residence, 6 dummies for city sizes. (IVs very similar to Grossman). No validity or strength tests.

Findings. In all 4 regressions, wages were positively correlated with better health. In 3 of 4, the correlations were significant at the 0.05 level 2-tailed test. “In contrast to whites, blacks show a much stronger wage effect and a significant positive effect of wife's education, with no other factors being significant. The issue of reverse causality between the wage and health is addressed via a simultaneous equations’ health-wage model. Contrary to expected, but consistent with previous findings, the structural model yielded an even larger wage effect.”

Comment. IVs not likely to be valid. Sample limited to only married people.

S26. Lee L-F. Health and wage: A simultaneous equation model with multiple discrete indicators. *International Economic Review*. 1982. 23 (1): 199-221.

[https://www.jstor.org/sS-table/2526472?casa_token=l8Jm36s4qVUAAAAA%3AaQrzB5G0wnLFc-k3R2_7YlysuMo-1JQHe50x7T4V82KEqhJOWRjrFolPomYwuwuB-VeQuJeQJlkak320O5OVv1aogx5CE4OVqfm_TaQ_FY1gDHdrS911kg&seq=1](https://www.jstor.org/stable/2526472?casa_token=l8Jm36s4qVUAAAAA%3AaQrzB5G0wnLFc-k3R2_7YlysuMo-1JQHe50x7T4V82KEqhJOWRjrFolPomYwuwuB-VeQuJeQJlkak320O5OVv1aogx5CE4OVqfm_TaQ_FY1gDHdrS911kg&seq=1) Accessed January 29, 2024.

Data: USA. 1966. Individuals. Cross-sectional. National Longitudinal Survey of Men ages 45-59, Survey year 1966. N= 2976.

Demographics: Men only, ages 45-59. Race is binary, white =1 and all others = 0. Presumably, Black, Hispanic, Asian, are included in “other race.”

Health. Two outcomes. General health measures subjective excellent, good, fair, or poor and indicator measures whether health limits amount or kind of work person can do. For overall, self-reported health, equals 1,2,3,4 with 4= excellent. This variable is put into ordered probit estimator.

Wage. Hourly rate of pay at current or last job in natural logarithm scale of cents per hour, 1966. Measured as current wage, no lag. Mean is 5.6 and standard deviation is 0.56.

Covariates. Age, years of schooling, married, measure of assets, work-experienced-squared, reside in SMSA (large city), reside in south, white=1 (no others).

IVs. Unique IV variables (instruments) included work experience squared, living in an SMSA, living in the South, and white=1. No tests for valid IVs or strong IVs

Methods: Ordered probit for general health (excellent…poor), and probit for binary health-limits-work variable. Maximum Likelihood estimator, not least squares. Unusual version of IV method.

Findings: Consistently finds wages improve health whether measured as overall (excellent…poor) or lack of health-work-limitation in four of four regressions. Significant at the 0.05 level in one and at the 0.01 level in three.

Comment: Lee’s paper is old but was pathbreaking. By today’s standards, likely Lee uses incorrect version of the IV method. In addition, the IV variables chosen are lousy as they could easily directly affect health. Lee also presents estimates of health on wages using a simultaneous equations model. In those days, many people did not understand that “simultaneous equations models” were just another version of instrumental variables.

S27. Leigh JP, Chakalov BT. Effects of wages on smoking prevalence using labor unions as instrumental variables. *Journal of Occupational and Environmental Medicine*. 2023. 65 (4): e234-239.

Data. USA. 1999-2005. Longitudinal. Panel Study of Income Dynamics. N = 8446 – 37,117 person-years.

Demographics. Employees, age 18-70. No race or gender restrictions.

Health. Current smoker, binary.

Wage. Hourly wage = annual earnings/annual work hours. The wage variable included in PSID measures annual earnings from work including wages-per-hour, salary, bonusses, overtime, tips, and commissions divided by annual work hours.

Covariates. Age, age-squared, gender, race/ethnicity, marital status, education, and indicator variables for private-sector job, regions, and years.

Method. Instrumental variables and ordinary least squares.

IVs. Union membership or covered by contract, binaries

Findings. Higher wages reduce smoking prevalence.

S28. Leigh JP, Du J. Are low wages risk factors for hypertension? *European Journal of Public Health*. 2012. 22 (6): 854-859.

Data. USA. Longitudinal. 1999-2005. Individuals. N= 17,295 person-years and N =5651 people.

Demographics. No gender or race/ethnicities exclusions. People must be employed. Ages 25-65.

Health. “Has a doctor ever told you that you have hypertension?” yes/no. Also, “time to hypertension”.

Wage. Hourly wages, i.e. annual wage earnings/annual work hours. Employees and self-employed.

Covariates. “Age, race, and gender …. Non-wage socio-economic and geographic variables included marital status, education, whether respondent had any medical insurance (including employer-provided, private or Medicare), whether self-employed, part-time employment, unemployed, salaried occupation, union member and four US Census, regions.”

IV? No IVs.

Method. Prospective since in the beginning (base years), no one reported hypertension; hypertension was diagnosed over the 7 years of the study. Logistic regressions with random effects and Cox proportional hazards regressions.

Findings. Statistically significant findings that higher wages reduce hypertension incidence especially for women and people aged 25-44.

S29. Lindeboom M, Kerkhofs M. Health and work of the elderly: Subjective health measures, reporting errors, and endogeneity in the relationship between health and work. *Journal of Applied Econometrics.* 2009. 24: 1024-1046.

Data. Netherlands. 1993, 1995. Longitudinal. “….first two waves of the Leiden University Center for Research on Retirement and Aging (CERRA) panel survey. It resembles the American Health and Retirement Study (HRS). …. fall of 1993 and consists of 4727 households in which the head of the household (defined as the main income earner) was between 43 and 63 years of age…... In each household both head and partner, if present, were interviewed. In … 1995 the same respondents were contacted for a second interview. Approximately 74% of the first wave respondents participated in this second wave, which resulted in about 3500 households. ……we focus on the male heads of households, excluding the self-employed…… After removal of inconsistent observations and observations with missing values on the key variables, we obtain a dataset of 3038 individuals.”

Demographics. Male, heads-of-households. Ages 43-63. No race/ethnic data. No self-employed included.

Health. “… 57 items of the Hopkins Symptoms Checklist (HSCL). The responses to these questions result in a total health score, which can take on an integer value between 0 (best health state) and 171(worst health state).” Authors also use a measure of health-limitations-at-work and disability benefits but do not use income to predict limitations.

Wage. “Mean of annual income associated with working until the mandatory retirement age”

Covariates. Age, whether partner, family size, education, number months ever worked in life. Disability benefits are not covariates for explaining HSCL (“health stock”).

IVs. No IVs. Random instead of individual fixed effects.

Findings. S-table IV. Dependent variable is HSCL (“health stock”). Wage-income negatively correlated with HSCL (and high scores of HSCL indicate poor health). Significant at the .10 level in 2-tailed test. “We also find that higher incomes are associated with better health.”

S30. Nocera S, Zweifel P. 1998. The demand for health: An empirical test of the Grossman Model using panel data. In: Zweifel, P. (eds) Health, the Medical Profession, and Regulation. Developments in Health Economics and Public Policy, vol 6. Springer, Boston, MA. 1998. <https://doi.org/10.1007/978-1-4615-5681-7_2>

Data. Switzerland. 1981 and 1993. Longitudinal. Data from “questionnaires sent to members of a Swiss sick fund years 1981 and 1993. In addition, information about the utilization of medical services in the years 1981 through 1992 and insurance records for the years 1987 through 1992 are available. Since the socioeconomic information (e.g., income, wealth, work, lifestyle) from these two sources covers only the years 1976 to 1980 and 1989 to 1992, the values for the years 1981 to 1988 had to be interpolated.” Two samples. N = 1908 and N = 2544.

Demographics. Men and women (= about 37%). Age limits not specified. Means and (standard deviations) from two samples: 40 (10), 41 (8). No race/ethnicity information. 98% of people in the labor force. “Since over 80 percent of the individuals live in the canton of Bern, they are assumed to face identical prices of medical services, permitting to omit this variable in estimation.” Presumably, the Swiss sick fund applies almost exclusively to working people, but authors do not explain whether retirees or homemakers or disabled people etc. are excluded.

Health. “The health stock of an individual is approximated by subjective health status (HEALTH), marked on a visual analog scale ranging from 0 (=very bad) to 4 (=excellent) in both questionnaires. In both samples, HEALTH averages 3 points. While Grossman (1972a) measured the stock of health in a similar way, Wagstaff (1986), Leu and Doppmann (1986), and Leu and Gerfm (1991) treated it as an unobservable variable.” “The demand for medical care is measured by annual gross health expenditure as reported by the insurer (MEDICAL) in Swiss Francs (1 Sfr R: 0.85 US$ at 1995 exchange rates). MEDICAL is zero in 30 percent of the observed person-years in both samples. In keeping with equations (9) and (9'), the logarithm of MEDICAL should be used.”

Wage. The wage rate (WAGE) is gross monthly income divided by monthly average hours of work. “Since around two percent of the individuals in both samples do not participate in the labor market, a wage rate of zero is reported for them.”

Methods. “The pooling of cross section and time series data calls for a modeling of individual effects either in form of fixed individual effects (using dummy variables) or random effects (by splitting up the error term. In order to save degrees of freedom and to avoid problems caused by time-invariant regressors, the random effects model was estimated using the generalized least squares (GLS) estimator available in LIMDEP.” There were 2 regressions explaining medical care use. The reduced form excluded health stock (HEALTH) as a covariate and the second, structural form, included the predicted values of HEALTH as a covariate.

Covariates. Age, education (vocational, college, university), female, live alone, smoker, considers self overweight, hours of weekly exercise.

IVs. No IVs for wages.

Findings. In one sample (N=1908) wages are positively and significantly correlated with HEALTH; in second sample (N= 2544) , wages are insignificant. Coefficients cannot be interpreted since HEALTH is log of 0,1,2,3,4. For the medical care use regressions, wages are never statistically significant.

Comment. Smoking, weight, and exercise are covariates, but wage likely affects them, too; they should be dependent variables. “Since around two percent of the individuals in both samples do not participate in the labor market, a wage rate of zero is reported for them.” NO ! this is terrible. Should omit them.

S31. Rodriguez DA, Targa F, Belzer MH. Pay incentives and truck driver safety: a case study. *Industrial and Labor Relations Review.* 2006. 59 (2): 205-225.

Data. USA. 1995-1998. Longitudinal. Individuals. N = “2,368 unscheduled over-the-road J.B. Hunt drivers who received pay increases during a period of up to 25 months between 1995 and 1998. J.B. Hunt is one of the three largest nonunion truckload trucking and logistics firms operating in North America. The data cover two one-year periods: September 1995-September 1996, and March 1997-February 1998, inclusive. ….16% of drivers left the firm while under observation, and we observed 71.6% of drivers for more than 12 months.”

Demographics. No restrictions on gender or race/ethnicity or age. 2.4% female, 73% white and 39% single.

Health. Crash involving >=$1000 (1998) regardless of who was at fault. 826 crashes observed. 74% of drivers did not have any crashes >=$1000.

Wage. Pay rate, cents-per-mile. From 28 cents to 38 cents per mile.

Method. Event study. Pre-post design. Before-and-after comparisons, i.e. crashes before pay raise in 1995-1996 and after in 1997-1998.

Findings. Crash incidence drops by >50%, see their figure 1.

Comment. Authors were not able to account for any possible underlying trend reducing crash rates over the two years. But any trend is unlikely to result in a 50% reduction. The dependent variable is “crash”, not “crash-with-injury,” i.e. the dependent variable is not a direct measure of health; might call it health behavior, however.

S32. Schmitz LL. Do working conditions at older ages shape the health gradient? *Journal of Health Economics.* 2016. 50. 183-197.

Data. USA. Longitudinal. 1992 – 2010 for the Health and Retirement Survey (HRS) and 1980 to 2008 for Social Security Administration’s (SSA) Master Earnings File (MEF). Matches were made based on age. “For example, a worker who entered the HRS in 1992 at age 51 was 39 in 1980 for the SSA-MEF file, while a worker who entered the HRS at age 51 in 1998 was 33 in 1980.” N = 7394 person-years.

Demographics. Age 50+ in 1992. Full-time male workers ages 50-65. No upper restriction. People followed until they drop out of sample (for whatever reason). Log of earnings measured in 1980 and average annual growth of earnings from 1980 to 1992.

Health. Self-assessed health. Excellent or very good = 1; good , fair, poor = 0. Also, “(1) doctor diagnosed heart disease, (2) doctor diagnosed high blood pressure or hypertension, (3) cognitive functioning (total word recall summary score), and (4) depression (Center for Epidemiological Studies-Depression (CESD) summary score).”

Wage. Wages are taken from W2 Box 1, or the “Wages, tips, and other compensation” in the Social Security Administration’s (SSA) Master Earnings File (MEF). 2010 dollars.

Causal model. No IV or DiD or discontinuity or propensity score. Longitudinal data on people and lagged measures of health, hypertension, heart disease, cognitive functioning, and depression.

Covariates. 5 Measures of working conditions ( e.g physical hazards, degree of control), smoker, exerciser, education, parents education, childhood health fair or poor, work hours, self-employment status, 3 occupation dummies, 8 industry dummies, age, race dummy, veteran status, marital status, health insurance, 4 regions, union status, number of children.

Method. Least squares, Probit, random effects.

Findings. Whereas earnings from 1980 is not statistically significant in predicting self-assessed health status from 1992 to 2010, earnings growth from 1980 to 1992 is significant and positive in its association with excellent or very good health (p< 0.05). Earnings in 1980 is a positive and significant (p < 0.01) predictor of good cognitive functioning from 1992 to 2010 whereas earnings growth is not. Neither 1980 earnings nor earnings growth predicts hypertension or heart disease or depression from 1992 to 2010. Quote: “a one percent increase in average annual earnings growth during peak earnings years, or between the ages of 23 and 49 depending on the cohort, is associated with a 17.8 percent increase in the probability of reporting “excellent” or “very good” health (S-table 4, Column 5). The effect persists even after adding household income flows after age 50 and fluctuations in income at baseline, indicating the trajectory of an individual’s earning history may be just as important as the overall level of earnings for health (see S-table A1).14 This finding is in line with research that has linked downward mobility or unsuccessful career trajectories during peak earnings years with poorer self-reports of mental health in men at age 50 (Tiffin et al., 2005).”

Comment. Lagged measures of health did not proceed measures of earnings. So poor health could reduce earnings before 1992, i.e. before measures of earnings. But does have measure of childhood health (fair, poor =1). Limited to male, full-time workers only. Quote : “An important insight gained from this research is that the cumulative or durable impact of working conditions is potentially more relevant than any contemporaneous outcomes to health later in life. Among these studies, only one examines a large sample of older workers. Using data on the longest-held occupation reported in the HRS, Gueorguieva et al. (2009) find that health problems do accumulate over the life course and are systematically different by occupation for older workers. However, it is not clear from this analysis whether working conditions are the primary driver behind occupational differences and if they continue to shape differences in health between occupations at older ages.”

# S33. Sedigh G, Devin RA, Grenier G , Armstrong CD. Revisiting the relationship between wages and sleep duration: The role of insomnia. *Economics and Human Biology.* 2017. 24. 125-139.

Data. Canada. 2005 and 2010. Repeated cross-sections. Individuals. “This study uses the Canadian General Social Surveys (GSS) − Time Use, for 2005 and 2010, which allows us to examine carefully how sleep decisions respond to wages within different economic contexts: one of growth and the other of recession. Respondents are asked to complete a diary listing all their activities over a 24-h designated day beginning at 4:00 am.” “6455 (4443 non insomniacs and 2012 insomniacs) in 2005 and of 4668 (3160 non insomniacs and 1508 insomniacs) in 2010.”

Demographics. People with jobs that reported wages. Ages 23-65. “In 2005, 30% of the sample had sleep problems (25% of males and 35% of females), and in the latter survey, 31% of the sample had sleep problems (28% males, 35% females). No full-time students.

Wage. “The main variable of interest, hourly wage rate, is created from the annual personal income reported by respondents, divided by 12 months, divided again by 4.3 (weeks in a month) and then divided by the number of hours usually worked at all jobs in a week.”

Health. Minutes of sleep.

Covariates. “…marital status, age, gender, health status, presence of young children, wage, and other income.” “….we also create an “other income” variable by subtracting personal income from total household income.” Catholic, Protestant, other (including none), union member, dummies for Canadian province, months of year, whether sleep was recorded on weekend

IVs. Yes, IVs. Union status, education, and industry dummies. F-tests confirmed these were strong instruments. The Wooldridge score test for overidentifying restrictions sometimes “indicates that we cannot reject the null hypothesis of no correlation between the instruments and the error term” but other times it does not.

Method. IV. “In addition to estimating Eq. (1) for everyone in our sample, we divide the sample into two groups: those reporting regular sleep problems (insomniacs) and those who do not (non-insomniacs). The grouping is based on the answer to the question in the GSS 2005: “Do you regularly have trouble going to sleep or staying sleep?”

Conclusions. “a ten percent increase in wages led, in 2005, to about a 11–12 min decrease in sleeping per week, and in 2010 to a reduction of 18 min by males (no response by females). Our findings are comparable to the small number of other papers that have looked at sleep duration and wages: Biddle and Hamermesh (1990) find that a 10 percent increase in hourly wages results in about a 14 min a week adjustment; Szalontai (2006) finds a 12 min a week adjustment.” There were larger effects on insomniacs and during economic downturns. Neither Biddle and Hamermesh nor Szalontai looked at insomnia.

S34. Sundberg G. Health, work-hours, and wages in Sweden. In: Zweifel, P. (eds) Health, the Medical Profession, and Regulation. Developments in Health Economics and Public Policy, vol 6. Springer, Boston, MA. 1998. <https://doi.org/10.1007/978-1-4615-5681-7_2>

Data. Sweden. 1991. Cross-sectional. Swedish Level of Living Survey (LNU) from 1991. Ages 18 - 76 years. N = 3322 (1660 women and 1662 men).

Demographics. Ages 18-76, employed with positive hourly wages and positive work-hours. Women and men. No race or ethnic distinctions.

Health. “Ill health,” self-assessed. Respondents asked: "How do you judge your present health condition? Is it good, bad or something between? Instead of having the values 1 (bad health, about 2% of sample), 2 (health is something between, 13%) and 3 (health is good, 84%) we have the values …..assuming that the distribution is standard lognormal.” Values for women and men combined: bad = 11, between = 4, good = 0.8

Wage. Wage-per-hour at the time of the interview.

Covariates. Age, age-squared, years of schooling, married, gender, monotonous work, noisy work, one-sided work, physically demanding work, psychologically strained work, screen work, vibrations at work, sweaty work, stressful work, big city, exercise, young children, old children, work experience, work experience-squared

IVs for wages. Work experience and work experience squared. F-tests reveals they are strong. Basmann’s over-identifying restrictions test has a low p-value and therefore accepts the null hypothesis: “The predetermined variables not appearing in any equation have zero coefficients.” See his S-tables 2-4.

Methods. Three equations: ill-health, wages, work hours. Three-stage least squares. IVs for ill-health include big city and exercise; IVs for work hours include young children, old children, work experience, work experience-squared. IVs for wages include work experience and work experience squared.

Findings. Three S-tables: men and women combined (S-table 2) , men and women separate and log-work-hours is used (S-table 3), men and women separate and work-hours (un-logged) is used (S-table 4). For women and men combined, wages are negatively correlated with ill-health, but not significant. For women and men separately, wages are negatively correlated with ill-health and significant at the 0.05 level. Sundberg also finds upward-sloping supply curves: wages and hours are positively correlated but significant in only 1-of-6 regressions. From abstract: “A low wage, for example, may force people to work more, and additional working hours may cause stress and increase health deterioration. A low wage may also influence a worker’s investment in health negatively, and hence health itself. With a higher wage people are able to have more leisure time, which can be used to improve the health of the individual.”

Comment. Quote from abstract above implies backward-bending labor supply curve yet he finds wages and hours to be positively correlated and 1-in-6 is statistically significant.

S35. Wagstaff A. The demand for health: Some new empirical evidence. *Journal of Health Economics*. 1986. Vol 5: 195-233

Data. Denmark. Cross-sectional. 1976. Danish Welfare Survey (DWS), “household survey of a randomly-selected, nationally representative sample of the 1976 Danish population between the ages of 20 and 70”. All persons in the labor force. N = 2243.

Demographics. Ages 20-70, in the labor force. Men and women. No indicators for race or ethnicity.

Health outcomes. Single composite measure which he calls HEALTH for physiological and psychological health combines. Wagstaff calls these measures of “non-chronic” health. Composites use “quartimax rotation” technique and principal components analyses. Measures included “hands shake easily”, “often poor appetite”, “often have insomnia”, “pain in back/loin”, “respiratory problems” , “prolonged colds”, “ability to climb stairs”, 19 measures in all. Wagstaff also looks at number of doctor visits, hospital stays, and number of complaints about medicine use. He also forms six Composite measures: mobility, mental health, respiratory issues, pain, and unnamed factors 5 and 6.

Wage. Measured as hourly wage in 1976. Also a LIFE WAGE variable which is the lifetime sum of expected hourly wages.

Covariates in health equation. Education (years schooling plus training before 1976 and during 1976 (separate variables)), job in healthcare, spouse education, spouse job in healthcare, gender, age, family size, married and/or cohabiting, job tenure, job is physically demanding, job is mentally demanding, exposure to extreme temperatures on job, GP doctor availability, number hospital beds per 1000 residents, GP costs, “initial assets.” Denmark had “free”, government-provided, medical care in 1976.

Methods. Maximum likelihood. And a version of IV. Not clear whether standard errors were corrected for IV estimation.

IVs. Regional unemployment, industry unemployment and, strangely, an indicator variable for CHRONIC ILL-HEALTH, which “equals 1 if respondent has health problems of a permanent character concerning fitness for work or health in other respects.” No tests for strength or validity.

Findings. S-tables 13 and 15, maximum likelihood estimates. Wages are positive predictors of better HEALTH ( e.g. coefficient = 0.007 S-table 13) and significant at the 0.01 level (e.g. given the t-value of 3.21) in both S-tables. Given the nature of his HEALTH measure, the numerical values of the coefficients cannot be pragmatically interpreted. In S-table 15, LIFE WAGE is positively and significantly (0.01) correlated with HEALTH. There were mixed but mostly insignificant findings for medicine, doctor visits and hospital stays.

Comment. IVs are likely not valid. Regional and industrial unemployment likely affect workers health and certainly CHRONIC ILL-HEALTH does. His measure of health is for non-chronic conditions.

S36. Wolfson M, Rowe G, Gentleman JF, Tomiak M. Career earnings and death: a longitudinal analysis of older Canadian men. *Journal of Gerontology: Social Sciences.* 1993. 48 (4): S167-S179.

Data. Canada.1979-1989. Longitudinal. Canadian Pension Plan and Quebec Pension Plan. 100% of Canadian paid labor force. 545,769 males who attained age 65 on or after September 1, 1979 and followed until Spring 1989. By 1979, the government Plans had been in existence for over 10 years. At least 13 years of earnings data were available for each person prior to age 65.

Demographics. Men age 65 in 1979.

Health. Mortality. Length of time lived after age 65.

Wage. Cumulative earnings covering at least 13 years.

Covariates. Age categories, marital status, “interrupted” versus “uninterrupted” work history.

IVs. None.

How to address reverse causality ? Authors excluded disabled; used average earnings “thereby minimizing the impacts of any acute health conditions; • excluded earnings in the year of retirement, thereby excluding years likely to have been affected by any critical health events; • disaggregated by, not just "controlled for," age at retirement and marital status; • controlled for the effects of chronic degenerative health effects to the extent they limit earnings by including in the analysis individual level trends in earnings relative to average wages; and • considered associations between earnings and mortality where the lags are quite long — earnings between ages 52 and the early 60s, and mortality between age 65 and 74 conditional on surviving to age 65.”

Method. Survival curves and ordinary least squares.

Findings. Higher earnings predict longer life expectancy (p<0.01). “Significant mortality gradients are found throughout the earnings spectrum. Substantively, the results cast doubt on the primacy of causal explanations such as "reverse causality" and "health selection" “ whereby poor health results in low earnings.”

Comment. Authors attempt to control for unemployment with indicator for “interrupted” versus “uninterrupted” work history.

S37. Woo J, Shook J, Goodkind S, Ballentine K, Engel R, Kim S, & Petracchi H. Do wage increases help? Wage increases and material hardships among low-wage hospital workers. *Journal of Human Behavior in the Social Environment*. 2023. 33(2): 198-211.

Data. USA. Cross-section. 2016. Authors collected own data from a hospital. N = 166. Hospital and union negotiated a raise for July 2016. Authors asked people about well-being before and after the raise. “…unionized service, technical, and clerical hospital workers.” Retrospective since questions asked in June 2017.

Demographics. Both genders, all races/ethnicities, all ages of employees. No restrictions, e.g. 79% women and 27% African-American.

Health . “Hardships were operationalized using four domains: 1) housing hardship (3 items; e.g., could not pay the full amount of rent or mortgage on time); 2) medical hardship (3 items; e.g., could not afford medical treatment); 3) food insecurity (2 items for adults and 1 for children; e.g., how often they worried about whether their food would run out before they got money to buy more); and 4) financial insecurity (3 items; e.g., I live from paycheck to paycheck). Each individual item was reported as a dichotomous variable. The items comprising each hardship domain were then summed and dichotomized as the presence (e.g., those who indicated one or more hardship in that specific area) versus the absence of each domain of hardship (e.g., those who indicated zero) to represent any housing, medical, food, and financial hardship respectively.”

Wage. One-time, union negotiated wage increase in July 2016. On average, wages increased from $16.57 to $18.01. Different occupations experienced different wage increases.

Covariates. “… gender, race/ethnicity, age, and educational attainment, hourly pay rates before and after the wage increase, and other work characteristics (i.e., full-time/part-time, overtime)”and number of children < 18 in household.

Method. Before-and-after comparisons of means of indicators of hardship using chi-squares. Event study.

IV? NO IV .

Findings. Increased wages resulted in fewer hardships and statistically significant.

Comment. Authors used simple mean comparisons and chi-square tests, not even least squares regression.

S38. Xu X. The business cycle and health behaviors. *Social Science and Medicine.*  2013. vol. 77(C), pages 126-136.

Data. USA. Multi-year Cross-sections. Overall, 1976-2005. Current Population Survey, CPS (1976-2005), the Behavioral Risk Factor Surveillance System, BRFSS (1984-2005) and the National Health Interview Survey NHIS (1976-2001). “The CPS is used to estimate the effects of economic activity on wage and working hours. The BRFSS and the NHIS are used in the second stage to estimate the effect of wage and working hours on health behaviors. Demographic variables are common to all three datasets. The sample is limited to males ages 25-55 with some college education or less. The total sample size of the combined CPS-BRFSS data for the time period of 1984-2005 is 967,594, while that of the combined CPS-NHIS data for the time period of 1976-2001 is 364,078.” N = 460,841 to 146,539.

Demographics. Males age 25-55 with < college degree. Non-disabled.

Health. In BRFFS: Whether smoker (0/1); whether smokes > 20 per day (0/1); any alcohol use in last 30 days (0/1), any binge drinking (0/1, >= 5 or more drinks per occasion in last 30 days, heavy drinker (0/1, >= 60 drinks in last 30 days), exercise = 1 if any physical activity or exercise in last 30 days. From NHIS, any doctor visits in last 12 months (0/1), >1 doctor visits in last 12 months (0/1).

Wage. Annual earnings/annual hours = Wages-per-hour from the CPS. But for BRFSS, “Real wage rates, hours of work per week, and employment in BRFSS are predicted by using estimates from CPS samples in the first stage.” CPS also provides hours at work per week and employment status.

Covariates. Age, married, single, other marital status, white non-Hispanic, Hispanic, black non-Hispanic, < high school, = high school, > high school (remember no college in sample), state and MSA unemployment rates, mix of employment across broad industries (e.g. % state employment in manufacturing) “Data on state cigarette taxes and prices from 1976 to 2005 are from the Tax Burden on Tobacco (Orzechowski & Walker, 2006). Data on state beer taxes come from various issues of the U.S. Brewers’ Association Brewer’s Almanac, while state beer prices during the time period of 1984-2003 come from the Cost of Living Index (COLI).” State vehicle miles travelled per capita, county-level road deaths, county-level air pollutants.

IVs. Instruments are 8 industry mix variables ( eg. % of state employment in manufacturing) , and 36 (!) interaction terms ( e.g. state unemployment x age category x % manufacturing) . 44 instruments. As a group, these are very strong as Xu’s F-tests demonstrate. Xu does not have any tests for validity, however.

Method. 2-sample, 2-stage IV. In the first part of the first stage, wages-per-hour and work hours and employment (0/1) are regressed on education , marital status, age, race/ethnicity and instruments which are 8 industry mix variables ( eg. % of state employment in manufacturing) , and 36 (!) interaction terms ( e.g. state unemployment x age category x % manufacturing) using only the CPS and years 1976-2001. In the second part of the first stage, the predicted values of wages, hours, and employment are generated from the coefficients in the first part using values on covariates from BRFSS and NHIS. The coefficients are provided in the appendix. In the second stage, these predicted values as used as IVs in regressions in which smoking, drinking etc are the dependent variables.

Findings. Increasing wages caused by economic expansions results in more smoking (current smoker, smokes > 10 cigs per day, smokes more than 20 cigs per day), no effects on binge drinking or heavy drinking or exercise or physician visits. Increasing work hours caused by expansions result in more smoking and less binge drinking, less exercise, and fewer physician visits. “The results in column 1 suggest that a one-dollar increase in real wage rate is associated with a 1.2 percentage point (3.5%) increase in smoking prevalence. In addition, a one unit increase in working hours per week (2%) is associated with a 0.8 percentage point increase in smoking prevalence.”

Comment. There are approximately 44 instruments, all of which likely affect health behaviors. Age affects exercise. Industry-of-employment affects drinking. Unemployment-within-region affects smoking and drinking. These instruments are not valid and Xu offers no tests for validity. Also, it is not clear how Xu treats wages for people without jobs. Xu states “For those who were not in the labor market, their usual working hours per work were defined as zero.” The sample sizes in the appendix are the same for all three variables ---wages, hours, and employment. This suggests Xu also assigns zeros to wages for people without jobs. (If Xu had restricted to only people with jobs for the wage and hours regressions, the sample sizes for wages and hours should be less than those for employment). In this “zeros” case, wages and hours are not just measuring wages and hours but also employment and unemployment. Also, why restrict to people with less than college ?

1. Studies that, at first, seemed to fit the criteria but were ultimately rejected. Note: Our “rejection” judgment reflects only our criteria for selection into this review concerning wages. Most are likely sound studies but just not focused on the effects of wages.

REJECT. Gerdtham Ulf-G, Johannesson M. New estimates of the demand for health: results based on a categorical health measure and Swedish micro data. *Social Science & Medicine.* 1999. 49 : 1325-1332.

Data. Sweden. 1991. Cross-section. Level of Living Survey. Ages 18-76 . Full sample = 5174. Employed sample = 3184.

Demographics. Ages 18-76. Men and women. No race or ethnic categories.

Health. “Assessment of own health on three-point scale ((0=poor health, 1=fair health, 2=good health).”

|  |
| --- |

Wage. Gross hourly wage in quartiles of wage distribution.

Covariates. Gender, dummies for age categories, BMI>30, unemployed, single, dummies for education, dummy indicating parents or siblings “had any health problems” dummies for family income quartiles, dummy for any taxable wealth, rural, small city, big city.

IVs . No IVs were used.

Method. Ordered probit for Health (= 0 or 1 or 2) .

Findings. Increasing wages correlated with better health (p<0.01).

Comment. REJECTED. No IVs or other modern causality techniques or long-run data.

REJECT. Grossman M. The Demand for Health: A Theoretical and Empirical Investigation. NBER.1972. <https://www.nber.org/books-and-chapters/demand-health-theoretical-and-empirical-investigation> Accessed March 22, 2024.

Data. USA. Two samples. 1. 1963. Cross-section. Individuals. University of Chicago NORC sample. “… whites in the labor force who reported positive sick time in 1963. This analysis is emphasized because of the 1,770 persons in the labor force, 558 had some sick days' in 1963 and 1,212 had no sick days.” 2. 1960. Second sample is aggregate data from 48 of 50 states in 1960 (Hawaii and Alaska data were not available). N = 48.

Demographics. In NORC, men and women. Whites only. In mortality data, men and whites only.

Health. Two variables. First is a measure of healthy days, i.e. 365 – number sick days off work for individuals. Second is the crude death rate of whites within states.

Wage. In NORC data, “wage rate”. For mortality data, wage is measured by median annual earnings for men (presumably all races) not weighted by average weeks worked because Grossman says average weeks worked are virtually the same across states.

Covariates. Family income, years of schooling, for both “health stock” and mortality. In addition, for “health stock”: family size, gender, and medical spending For mortality: price of “paramedical personnel”, price of cigarettes, expected death rate based on age.

IVs. No IVs for wages.

Method. Ordinary Least Squares and Two-stage Least Squares (the latter assuming medical care spending is endogenous).

Findings. Wages are positively and significantly correlated with both good health and lower mortality.

Comment. REJECTED. No causal methods for wages.

REJECT. Smith JP. Healthy bodies and thick wallets. The dual relation between health and economic status. *Journal of Economic Perspectives.* 1999. 13 (2). 145-166. Data drawn from S-table 4-15 in Smith, James P. and Raynard Kington. 1997. “Race, Socioeconomic Status and Health in Late Life,” in *Racial and Ethnic Differences in the Health of Older Americans.* Martin, Linda, and Beth Soldo, eds. Washington, DC: National Academy Press, pp. 106–62

Data. US. Cross-sectional. 1992. Health and Retirement Survey , HRS.

Demographics. Ages 51-61 in 1992. Both genders and all races and ethnicities.

Health. Self-reported: excellent, good, fair, poor.

Wage. Weekly wages. No adjustment for hours worked.

Covariates. Retirement income, welfare income (for both respondent and spouse, if any), household assets and wealth tercile, black, Hispanic, female, and years of schooling.

IVs. No IVs.

Method. Ordered probit because dependent variable is excellent, good, fair, poor.

Findings. Wages are positively and significantly correlated with good health when employment status is not controlled for. When employment status is controlled for, wages are no longer significantly correlated with health (either good or bad). Smith suggests that it is employment/unemployment , not wages that are the true cause of the correlation between wages and health and that , likely, good health allows the person to hold a job so that causality runs from good health to wages.

Comment. Only for ages 51-61. Cross-sectional data and no causal inference.

REJECT. Hartwig J, Strum J-E. Testing the Grossman model of medical spending determinants with macroeconomic panel data. *The European Journal of Health Economics*. 2018. 19: 1067-1986.

Data. Country-level data from 12 to 29 (depending on the regression-specification) OECD countries, 1970-2010.

Demographics. Aggregate data across countries , therefore, all genders and races and ethnicities and age groups.

Health. Medical expenditures per capita, adjusted for inflation and exchange rates.

Wage. “Employee compensation” which, in the US, includes health insurance.

Covariates. Percent of the population >= 65 years, and <= 4 years old, mean years of schooling, price of medical care, traffic accidents per capita, alcohol consumption per capita, tobacco consumption per capita, population density , female labor force participation rate, life expectancy at age 65, GDP per capita, total government spending per capita, unemployment rate, percent of population with health insurance coverage and other measures of medical care, e.g physicians per capita.

IVs. No IVs

Method. Ordinary Least Squares, two-way fixed effects for years and countries. Difference-in-differences.

Findings. Wages (employee compensation”) are strongly and positively correlated with medical spending per-capita.

Comment. In the US and some other countries (but not in others), “employee compensation” includes health insurance . REJECTED because dependent variable is spending and spending is not a direct measure of health

REJECT Maczulskij T, Haapanen M, Kauhanen A, Riukula K. Decentralized wage bargaining and health. *Economics & Human Biology*. 2024 Dec 1;55:101433.

Data. Finland. 2005-2013. Five data sources. “Harmonized Structure of Earnings Statistics (HSES) data from Statistics Finland, which contain individual and firm identifiers…..The HSES data are representative and include private sector firms (with more than four employees) and their employees annually from 1995 onwards.” “We add job tenure information to the HSES data for all Finnish employees obtained from the FOLK register of Statistics Finland.” “We match these data to those collected by Kotilainen (2018: 66–69) from each private sector collective agreement and supporting documents for the period 2005–2013. The collective agreement data include information on the magnitude and timing of wage increases stipulated by the contracts.” “For our purposes, the most important information concerns whether the contract includes a local wage increase allowance (i.e., a local pot)”. “Finnish Hospital Discharge Register (HDR), compiled by the Finnish Institute for Health and Welfare.” “…. population register data on sickness absence spells between 2004 and 2016 from the Social Insurance Institution of Finland (Kela). Kela records spells of absence that last longer than the waiting period of nine full working days.” “ Kela data on the reimbursed medications related to mental health disorders that were dispensed by Finnish pharmacies over the 1995–2016 period.”

Demographics. No restrictions on race or gender. Ages 18-64.

Health. 4 variables. 1. “….indicator variable that denotes whether an individual has experienced a spell of sickness absence lasting longer than nine full working days in a given year.” 2. “The second measure takes a value of one if an individual has at least one mental health-related medicine purchase per year, and zero otherwise.” 3. “We create a third indicator variable denoting whether an individual has been on a sick leave spell caused by mental health disorders.” 4. “The fourth variable denotes whether an individual has been hospitalized due to mental health disorders in a given year and zero otherwise. Hospitalizations are quite uncommon, as they include only severe mental health disorders.” For our purposes, we focus on absence as drugs and hospitalizations can be influenced by the income effect and therefore not “pure” measures of health.

Wage. Hourly earnings = monthly earnings/monthly hours. And : “We characterize the decentralization of collective bargaining by an indicator variable that takes the value of one if the collective agreement has the possibility of a local wage increase allowance (local pot) in a given year. The reference category is collective agreements without local (i.e., firm-level) wage increase allowances.”

Covariates. “….age (five categories), tenure (five categories), marital status, part-time work, and occupation (two-digit ISCO classification). The regression models include firm-level controls for firm size (five categories) and 15 industry indicators.”

Methods. Longitudinal with fixed effects for individuals. Least squares, linear probability model.

IVs. No IVs.

Findings. In their “highlights” section, authors claim “Local wage increases improve mental health in white-collar intensive firms.” “Local wage increases reduce mental health in firms where blue-collars dominate.”

Comment. “Local wage increases” act as a kind of minimum wage. “Local wage increases” are associated with higher earnings only for white-collar, not blue-collar workers. For blue-collar workers, “local wage increases” are associated with higher unemployment. Since we are only concerned with wages for workers in our paper, we ignore the blue-collar result pertaining to employment and conclude that these Maczulskij et al (2024) findings imply that higher earnings result in fewer mental health medicines for white-collar workers but have no effects for blue-collar workers. We found only one, weakly (0.10) significant correlation among white-collar workers and this was for purchases of mental health drugs, not for absenteeism. But since we do not include drugs purchases in this review, we conclude that Maczulskij et al (2024) find only insignificant effects for wages on health which they measure as absenteeism. REJECT because the only relevant variable for our purposes was absenteeism and we decided to exclude all studies of absenteeism because most of these studies do not distinguish between absence due illness versus all other reasons for absence.

REJECT. Galama TJ, Hulligie P, Meijer E, Outcault S. Is there empirical evidence for decreasing returns to scale in a health capital model ? *Health Economics.* 2012. 21. 1080-1100.

Data. USA. Longitudinal. Individuals. 2003 and 2007. Panel Study of Income Dynamics (PSID). N=5483 in 2003, N= 5594 in 2007.

Demographics. Men only. All races and ethnicities.

Health. Authors also investigated an aggregate “health” variable ---a composite of subjective ratings (excellent…poor), activities of daily living, smoking and others--- but they did not allow total their measure of wages to enter as a covariate for their “health” variable. Authors also look at medical expenditures, out-of-pocket medical expenditures, nights in hospital. The PSID does not have total medical expenditures or out-of-pocket spending for individuals (PSID does have some medical spending data on households). Authors therefore predict medical expenditures using data from the Medical Expenditure Panel Survey. The same set of covariates is used to match MEPS and PSID samples. The expenditures from the MEPS samples are then assigned to the PSID based upon these matches.

Wage. Log of hourly wage.

Covariates. Whether private insurance, age, education, spouses’ education, black, other race, married, suburban, rural, household size,

IVs. No IVs for wages.

Method. Least squares, IV, Poisson, probit, negative binomial, double-hurdle. Longitudinal data with random effects.

Findings. Wages are not significantly correlated with any measure of spending i.e., out-of-pocket costs, or total medical spending, nor with nights in hospital.

Comment. REJECTED. For the regressions that include wages, the authors also enter their composite health measure as a covariate. This technique therefore does not allow a “clean” test of the effects of wages on health. And wages are not the focus of this article. Dependent variable is medical services, not a direct measure of health.

# REJECT. Otten JJ, Bradford VA, Stover B, et al. The culture of health in early care and education: workers’ wages, health, and job characteristics*. Health Affairs (Millwood).* 2019. 38 (5): 709-720.

Data. US. Cross-sectional. Individuals. 3 sites: Seattle, King County Washington, and Austin. 2017. N = 336 . Authors collected data. Retrospective data collected by authors.

Demographics. Child-care workers. Employed, no special age restrictions. Mean age = 37. No gender or race/ethnic restrictions. Females = 341, males = 22.

Health. “The 12-Item Short Form Health Survey (SF-12) was used to assess both physical and mental well-being.”

Wage. Binary, either >=median for worksite or < median.

IV? None.

Method. Differences in means. T-tests.

Findings. Lower wages were associated with poorer mental health and higher food insecurity.

Comment. REJECTED. No causal methods; simply t-tests. No long-run or longitudinal data.

REJECT. Clougherty JE, et al. Workplace status and risk of hypertension among hourly and salaried aluminum manufacturing employees. Social Science & Medicine. 2008. <https://www.ncbi.nlm.nih.gov/pmc/articles/PMC2659853/> . The propensity score separates people by blue-collar vs white-collar, not via job grade. Also, income (which includes individual’s wages) is included as a covariate .

REJECT. Bala MM, Singh S, Kumar N, Janor H. Predicting key drivers for health care expenditure growth in the Middle East region: a Grossman-PLS modeling approach. *Expert Review of Pharmacoeconomics & Outcomes Research* 2022. 22:6, 1021-1031, DOI: 10.1080/14737167.2022.2073222 .

Data. Longitudinal. 15 Middle Eastern countries, 2000-2016. Annual aggregate data. N= 255.

Demographics. Annual country-wide data on entire population. No restrictions on age, race, gender, employment.

Wage. Country-wide average “compensation for employees.”

Health. Healthcare spending per capita (US dollars, purchasing power parity) and healthcare spending as percent of GDP.

Covariates. Percent of population > age 65, physicians per 1000 residents, hospital beds per 1000 residents, nurses and midwives per 1000 residents, R&D expenditures as percent of GDP, average years of education.

IVs ? No

Method. OLS with 2-way-fixed effects, therefore difference-in-differences.

Conclusions. Higher wages predict higher spending. Significant at the 0.01 level. REJECTED because dependent variable is spending, not a direct measure of health.

REJECT. Erbsland M, Riued W, V Ulrich. The Impact of the Environment on the Demands for Health and Health Care: An Empirical Analysis for Germany. In: Zweifel, P. (eds*) Health, the Medical Profession, and Regulation. Developments in Health Economics and Public Policy, vol 6.* Springer, Boston, MA. 1998. <https://doi.org/10.1007/978-1-4615-5681-7_2>

Data. West Germany. Cross-sectional. 1986. Third wave of the West German Socio-economic Panel (SOEP), collected in 1986. Employees only. “We confine the analysis to the working population, since the use of the duration of sick leave as health indicator makes sense only for working individuals.” N = 4874

Demographics. Men and women. No race or ethnic distinctions. Age limits never specified.

Health. Health Indicators: “handicapped individuals 1-3 “, “chronic complaints”, “self-rated health 1-11” , days of sick leave. These health indicators are combined to form a composite variable, “health capital,” which is a “latent variable.” They use factor analysis to create this health capital variable. Medical care use: number of visits to GP, number of visits to specialists, hospital days in 1985.

Wage. “net monthly income”

Covariates. Gender, age, nationality, “education 1-3”, “doing sports 1-4”, private insurance, “community size 1-7”, “accessibility of resident physician 1-4”

IVs for wage. None

Method. Maximum likelihood. No version of IV.

Findings. In S-table 2, structural equations estimating direct effects, net monthly income is positively and significantly (<0.05) related to the composite health variable, negatively and significantly (<0.05) related to visits to GP, positively and significantly related to visits to specialist, and insignificantly related to hospital days. S-table 3 estimates indirect effect of income on medical care use (via direct effect of income on better health) and total effect (combines direct and indirect effects) of income on medical care use. Income has negative and significant effects on GP visits for both indirect and total effect; negative and significant indirect effects but insignificant total effects on specialists visits on hospital days.

Comment. Erbsland et al, in their empirical work, refer to “net monthly income,” not wage. They never clearly define net monthly income. Nevertheless, they restrict the sample to “employees only,” and their Grossman-modified theoretical model mentions wage as an important independent variable. In their key equation 32, wage appears as a determinant of medical care use but income does not. So likely Erbsland views “net monthly income” as a wage. Cross-sectional data and no causal models.

REJECT. Bolin K, Jacobson L, Lindgren B. The demand for health and health investments in Sweden 1980/1981, 1988/89, and 1996/97. In Lindgren B. Individual Decisions for Health . Routledge and Taylor & Francis, London UK. 2002. <https://books.google.com/books?hl=en&lr=&id=WgeCAgAAQBAJ&oi=fnd&pg=PA93&ots=8UQVosFVYs&sig=1wnnPJT0JaIopKi1KO7A6AQ2iu8#v=onepage&q&f=true> Accessed March 21, 2024.

Data. Sweden. Longitudinal (but they only use wage from the middle year, 1988/89). Three years, 1980/81, 1988/89, 1996/97. Swedish biannual survey of living conditions (ULF acronym for words in Swedish). Begin with approximately 16,000 people, ages 18-84. But only about 40% followed all years. N = 3800 people in all years, 1980/81, 1988/89, and 1996/97. No race/ethnicity. Men and women included.

Health. Subjective, 1,2,3 with 1 is worse and 3 is best health. Exercise, with 1,2,3,4,5. 1 = never exercise and 5 = exercise at least twice a week.

Wage. Continuous wage rate measured in 1988/89 only, however. SEK-per-hour.

Covariates. Age, years of schooling, income from capital in SEK per year, married or co-habiting, divorced, child (= 1 if had child between 1980 to 1989), gender.

IVs. No IVs.

Method. Ordered probit for health (re-scaled to 0,1,2) and exercise ( re-scaled to 0,1,2,3,4)

Findings. Wages are negatively and significantly correlated with poor health and positively and significantly correlated with good health (both p<0.10). No statistically significant effects on exercise.

Comment. They could have looked at the effects of changes in wages on health and exercise but they did not. This is essentially a cross-sectional analysis. Just correlations, no IVs or difference-in-differences or other causal models.

REJECT. Wagstaff A. The demand for health: An empirical reformulation of the Grossman model. *Health Economics.* 1993. Vol 2 : 189-198. <https://onlinelibrary.wiley.com/doi/epdf/10.1002/hec.4730020211> Same paper as appears in edited volume by Andrew M. Jones and Owen O’Donnell. *Econometric Analysis of Health Data*. John Wiley & Sons . REJECT! Wagstaff uses household income, not wages for one person.

Data. Denmark. 12 months of longitudinal data, Oct 1982 – Sept 1983. “ Danish Health Study (DHS). This followed some l000 households (1752 adults) over a period of 12 months, beginning October 1982. Data on health and background variables (such as education) were obtained at the beginning and end of year, and detailed information on morbidity and use of health services was obtained for each week of the study by means of a diary.”

Demographics. Author is not clear. “The empirical model has been estimated for all adults, irrespective of whether or not they are in paid employment.” Likely similar to earlier Wagstaff study using the Danish Health Study which included people ages 20-70 in the labor force, men and women, but no race or ethnicity recorded.

Health. 2 variables. Overall health and medical care use. Health measured as a composite from answers to questions: 1. involving whether person has a “functional limitation” that limits anything they “want to do”, 2. “according to doctors I have seen, my health is now excellent” and person responds with “agree”, “don’t know”, “disagree” and “entirely disagree” and 3. Overall, self-rated health (excellent….poor). Medical care is a composite measure from 1. Visits to general practitioner doctor , 2. number of days in a hospital, 3. number of visits with a specialist doctor , 4. number of sessions with a physiotherapist, 5. number of outpatient visits, 6. number of visits to an emergency room.

Wage. “… equivalent household pre-tax monthly wage income in October 1982.”

Covariates. Not many. Years of schooling, gender, age. But also composite health in in 1982.

Method. Maximum likelihood. Wagstaff unique approach uses the 1982 health measure as a covariate in predicting the 1983 health measure. Similar to first differences.

IVs. None.

Findings. Wages are positively, but not significantly, correlated with health for people <= 41 years old (S-table 2a). For people > 41 years old, wages are positively and significantly correlated with health (S-table 2b). Wages are not significant in any regression for medical care use. Numerical values of coefficients cannot be interpreted because the composite health measure cannot be interpreted.

Comment. Only years of schooling, gender, and age enter as covariates into the health equation. Since Wagstaff’s wage variable is for household income, he should at least have entered married as a covariate. Better yet, define wage as applying only to one person, not the household. Wagstaff apparently does not think this is necessary since he has 1982-health as a covariate; not clear why. Although Wagstaff has data on doctors-per-capita and beds-per-capita, he does not use them as covariates to help explain his composite health variable. No insurance variables are covariates, but these are not necessary because Denmark had universal insurance at the time the data were collected. Uses household wages, not wages of one or the other spouse and there are no controls for marital status.

REJECT. Waldron H. Trends in mortality differentials and life expectancy for male Social Security-Covered workers, by socioeconomic status. *Social Security Bulletin.* 2007. 67 (3): 1-28. Measure of earnings is too crude. She compares men’s earnings in the bottom half of the earnings distribution to men in the top half. She is measuring relative, not actual, earnings and in the crudest possible way: bottom half versus top half. No other study we considered used relative wages or earnings. In addition, she does not control for other determinants of health such as education. In fact, she explicitly states that her measure of earnings is a proxy for socioeconomic status. She nevertheless finds strong evidence that lower long-run earnings are associated with shorter life expectancy.

REJECT. Milner, A., Aitken, Z., Kavanagh, A., LaMontagne, A.D. and Petrie, D., 2016. Persistent and contemporaneous effects of job stressors on mental health: a study testing multiple analytic approaches across 13 waves of annually collected cohort data. *Occupational and environmental medicine*, *73*(11), pp.787-793.<https://oem.bmj.com/content/73/11/787.short?casa_token=uLUpAAAnFwwAAAAA:OHCmbEDJuxxgk-xM2I-XyD5WUyLaUt_jwGvCxvKD-zZQKtn_APG2xAXVynwW5xR_5WPcP8UmHuMlDQ> panel data, individual fixed effects and first diffs, “unfair pay” predicted mental health. Not sure how to interpret “unfair pay”.

REJECT. Maclean, J.C., Webber, D.A., French, M.T. and Ettner, S.L., 2015. The health consequences of adverse labor market events: Evidence from panel data. *Industrial Relations: A Journal of Economy and Society*, *54*(3), pp.478-498.<https://onlinelibrary.wiley.com/doi/full/10.1111/irel.12099?casa_token=3qIQ242xzawAAAAA%3AwOkWNR3sgVDciO_EV1mJ0BW1dmvxg3H8dJrIYToxpUNQdnqKb6o45sHrPAam2yWYxreFcrm9QmVLZM6gag> PERCEIVED financial harm is key independent variable, not wages. They find “Our findings suggest problems with coworkers, employment changes, and financial strain are associated, respectively, with a 3.1 percent (3.3 percent), 0.9 percent (0.2 percent), and 4.5 percent (5.1 percent) reduction in mental health among men (women). Associations are smaller in magnitude and less significant for physical health.”

REJECT. Dupere V, Beauregard N, Pelletier-Dumas M, Racine E, Tardif-Grenier K. Employment wages and diseases of despair in early adulthood: Links through subjective socioeconomic status and cumulative stressor exposure. *Social Science & Medicine: Mental Health* 2024. 5. Number 100324.

Data. Canada. Longitudinal. Individuals. 2012-2023. In the years 2012-15, N = 543; years 2016-2019, N= 384; years 2020-2023, N = 302.

Wage. “… a “current wage” variable was derived representing the wages of the main job (e.g., if a participant had a full-time job and a part-time job, the wages of the full-time job were retained) held at the time of the interview. Those not working were assigned a wage score of 0.” This will not work! Unemployment is not simply wage = 0.

REJECT. M.-H. Kim, C. Kim, J.-K. Park, and I. Kawachi, “Is precarious employment damaging to self-rated health? Results of propensity score matching methods, using longitudinal data in South Korea,” Social Science & Medicine, vol. 67, no. 12, pp. 1982–1994, Dec. 2008, doi: 10.1016/j.socscimed.2008.09.051. But precarious employment included wage as only 1 factor, i.e. precarious employment means more than just low wages. No analysis of effects of wages alone on health.
